# A novel virulent *Litunavirus* phage possesses therapeutic value against multidrug resistant *Pseudomonas aeruginosa*

**DOI:** 10.1038/s41598-022-25576-6

**Published:** 2022-12-07

**Authors:** Varintip Lerdsittikul, Metawee Thongdee, Somjit Chaiwattanarungruengpaisan, Thassanant Atithep, Sukanya Apiratwarrasakul, Patoo Withatanung, Martha R. J. Clokie, Sunee Korbsrisate

**Affiliations:** 1 Veterinary Diagnostic Center, Faculty of Veterinary Science, Mahidol University, Nakhon Pathom, Thailand; 2 The Monitoring and Surveillance Center for Zoonotic Diseases in Wildlife and Exotic Animals, Faculty of Veterinary Science, Mahidol University, Nakhon Pathom, Thailand; 3 Frontier Research Center, Vidyasirimedhi Institute of Science and Technology, Rayong, Thailand; 4 Department of Immunology, Faculty of Medicine Siriraj Hospital, Mahidol University, Bangkok, Thailand; 5 Department of Genetics and Genome Biology, University of Leicester, Leicester, UK

**Keywords:** Microbiology, Molecular biology

## Abstract

*Pseudomonas aeruginosa* is a notable nosocomial pathogen that can cause severe infections in humans and animals. The emergence of multidrug resistant (MDR) *P. aeruginosa* has motivated the development of phages to treat the infections. In this study, a novel *Pseudomonas* phage, vB_PaeS_VL1 (VL1), was isolated from urban sewage. Phylogenetic analyses revealed that VL1 is a novel species in the genus *Litunavirus* of subfamily *Migulavirinae*. The VL1 is a virulent phage as no genes encoding lysogeny, toxins or antibiotic resistance were identified. The therapeutic potential of phage VL1 was investigated and revealed that approximately 56% (34/60 strains) of MDR *P. aeruginosa* strains*,* isolated from companion animal diseases, could be lysed by VL1. In contrast, VL1 did not lyse other Gram-negative and Gram-positive bacteria suggesting its specificity of infection. Phage VL1 demonstrated high efficiency to reduce bacterial load (~ 6 log cell number reduction) and ~ 75% reduction of biofilm in pre-formed biofilms of MDR *P. aeruginosa*. The result of two of the three MDR *P. aeruginosa* infected *Galleria mellonella* larvae showed that VL1 could significantly increase the survival rate of infected larvae. Taken together, phage VL1 has genetic and biological properties that make it a potential candidate for phage therapy against *P. aeruginosa* infections.

## Introduction

*Pseudomonas aeruginosa* is a Gram-negative environmental saprophyte that colonizes and persists in natural environments such as plants, soil, and water^1,2^. It can cause infections in humans and animals. *P. aeruginosa* is a well-known cause of severe and potentially life-threatening nosocomial infections in humans and septicemia in burn victims^3^. *P. aeruginosa* is a zoonotic and has reverse zoonotic transmissibility^4^. Animals susceptible to *P. aeruginosa* infections include cattle, swine, horses, and companion animals such as cats and dogs^5,6^. *P. aeruginosa* infections in animals are associated with otitis externa, chronic deep pyoderma, wound and urinary tract infections^7^. Antibiotics are the drugs of choice for the treatment of *P. aeruginosa* infections; however, owing to their adaptive strategies and a variety of antibiotic-resistance mechanisms, multidrug resistant (MDR) *P. aeruginosa* has emerged. *P. aeruginosa* is among the six most common and serious MDR pathogens within the acronym “ESKAPE,” comprising of *Enterococcus faecium*, *Staphylococcus aureus*, *Klebsiella pneumoniae*, *Acinetobacter baumannii*, *Pseudomonas aeruginosa* and *Enterobacter* spp.^8^. Additionally, *P. aeruginosa* has the ability to form biofilms, which can protect these bacteria from antibiotics and promote long-term bacterial adherence on surfaces of medical devices^9^ and food industry equipment^10^. Hence, there is a pressing need to identify alternatives to antimicrobial drugs to combat MDR *P. aeruginosa* and its biofilm formation.

To address these problems, bacteriophages (or phages), a group of viruses that infect specific bacterial cells, have been reported as a potential alternative strategy for combating MDR infections. They do not affect the normal microflora and are characterized by relatively safe and easy augmentation^11–13^. Studies on phage therapy to treat antibiotic-resistant *P. aeruginosa* infections in human medicine are quite extensive^14,15^. For example, Roach et al.^16^ reported successful phage therapy for pulmonary *P. aeruginosa* infection which required synergy between phages and host innate immune components. However, to date, there have been relatively few studies^17,18^ aiming for veterinary applications, particularly in companion animals.

At present, despite phages specific to *P. aeruginosa* having been isolated worldwide, there has been little focus on phages isolated from Southeast Asia. Furthermore, although many isolated phages share 83–97% nucleotide sequence identity, they exhibit large variations in several phenotypic properties, such as their host range or efficient host lysis^19^. Phages isolated from one country/region may not effectively kill bacteria from other countries/regions. Therefore, there has been a tendency to focus on locally isolated phages. In addition, it is important to isolate new phages to accumulate information for the development of effective phage cocktail therapy.

In this study, a novel *Pseudomonas* phage, designated vB_PaeS_VL1 (referred to herein as VL1), was isolated from an urban sewage sample collected in Bangkok, Thailand. In vitro and in silico including virion morphology, the host range of infection and a whole genome sequence analysis were assessed. The ability of phage VL1 to destroy bacterial biofilms produced by MDR *P. aeruginosa* strains isolated from animal infections and the therapeutic efficacy in the *Galleria mellonella* model of infection was evaluated. Data generated from this study indicated that phage VL1 could be a valuable candidate for further development as a therapeutic or biocontrol agent against MDR *P. aeruginosa* infection.

## Results

### Isolation and characterization of phage vB_PaeS_VL1

**Figure 1:**
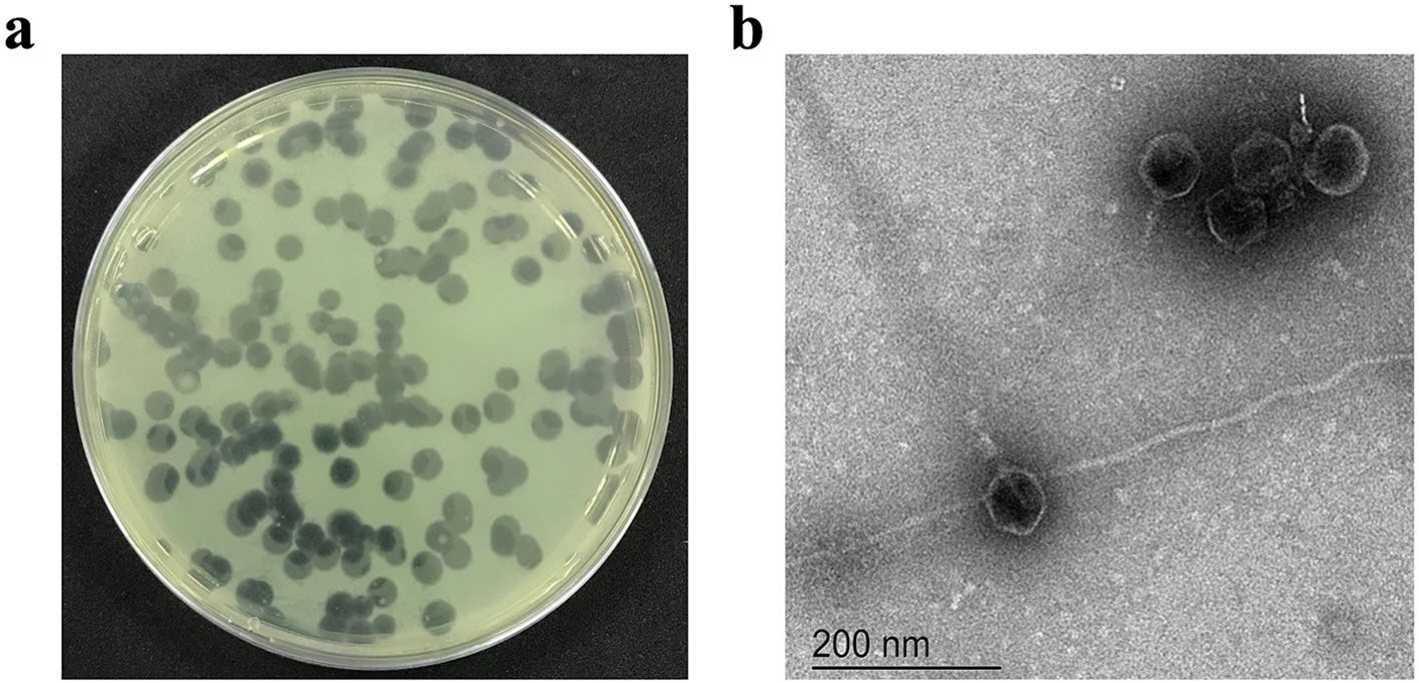
Plaque and morphology of the phage vB_PaeS_VL1. (**a**) Plaque morphology of phage VL1 formed in double-layer agar plate with *P. aeruginosa* ATCC27853 as host strain. (**b**) Transmission electron micrographs of phage VL1 negatively stained with 2% (w/v) uranyl acetate. The scale bar represents 200 nm.

*Pseudomonas* phage vB_PaeS_VL1 (VL1) was isolated using *P. aeruginosa* ATCC27853 as the host strain. On the lawns of *P. aeruginosa* ATCC27853, phage VL1 produced large, clear, and round plaques with a range of size 4–5 mm in diameter (Fig. 1a). TEM analysis showed that phage VL1 possesses an icosahedral head of approximately 61 nm in diameter and a short tail with an approximate length of 20 nm (*n* = 5 phages) (Fig. 1b), indicating that it is a member of the order *Caudovirales,* which features tailed viral particles and has a podovirus morphology.

### Genome analysis indicated that vB_PaeS_VL1 is a virulent phage and would be a safe therapeutic agent

Phage genome analysis is an important preliminary step towards the development of phage therapy. The genome of VL1 is 73,308 bp with a total 54.7% G+C content which is lower than that of *P. aeruginosa* ATCC27853 (G+C 66.12%). To identify whether the VL1 phage genome is linear or circular, PhageTerm^20^ analyzed the VL1 genome sequence (accession number OK665488). The result suggested that the VL1 phage has a linear genome. In addition, the presence of short direct terminal repeats (DTRs) of approximately 659 bp in length on the VL1 genome suggests that VL1 used a packing mechanism similar to the T7 phage (short DTRs) model (Supplementary data, Fig. S1). Furthermore, VL1 genomic DNA digestions with *Apa*I (single site on the phage genome) and *Nde*I (four sites on the phage genome) showed the presence of 2 and 5 DNA fragments, respectively, confirmed that VL1 has a linear genome (Supplementary data, Fig. S2).

**Figure 2:**
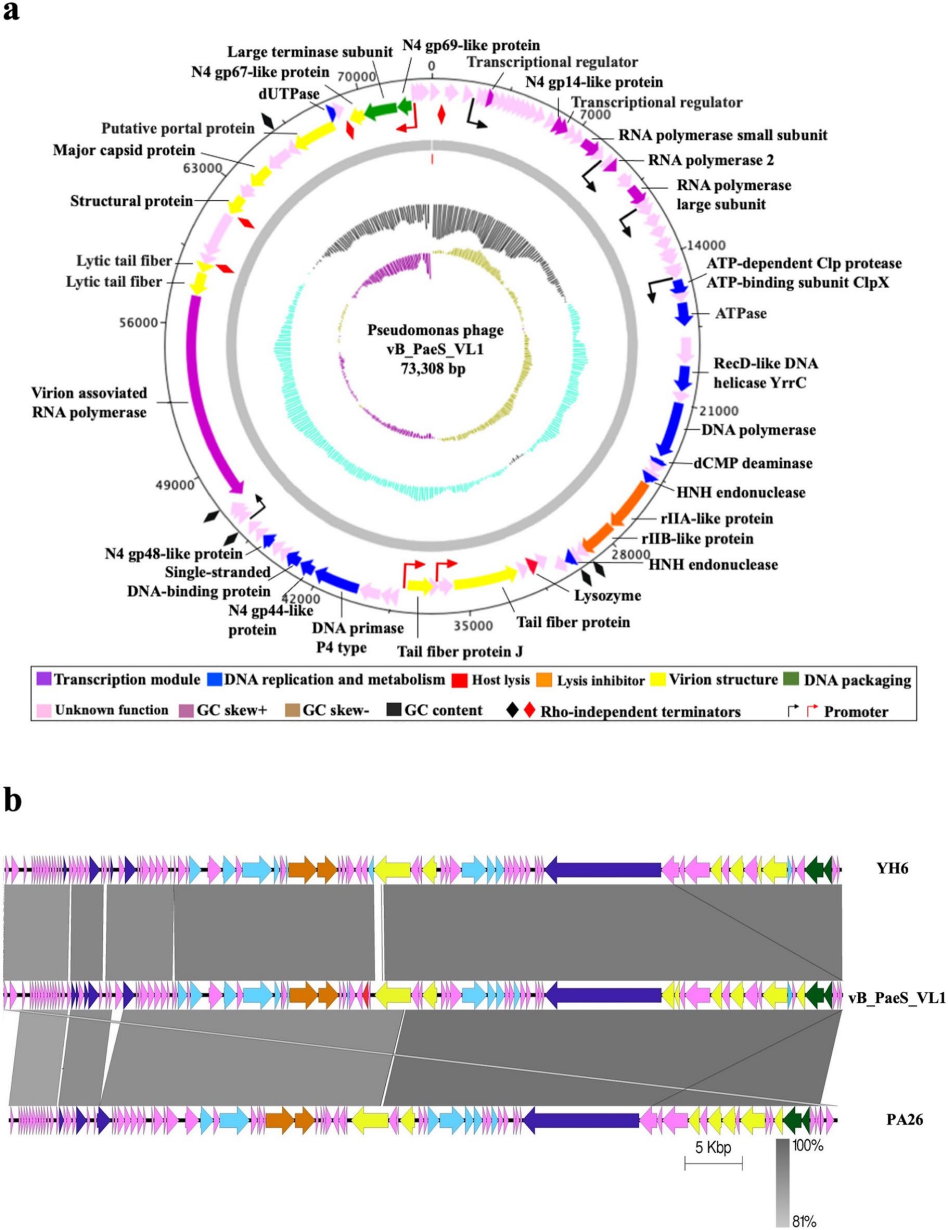
Phage vB_PaeS_VL1 whole genome analysis. (**a**) Map of genome organization of phage VL1. The outer ring shows a total of 92 open reading frames (ORFs) with their transcription direction and highlighted with different colors according to their functions. This circular map was generated using Artemis version 17.0.1. (**b**) Comparison of the whole genome sequences of the phage VL1 with similar phages (*Pseudomonas* YH6 and PA26). The colored arrows indicated ORFs according to their predicted function. The homologous regions between phages are indicated by gray shading. (Scale = base pairs).

Annotation of phage VL1 sequence revealed the presence of 92 putative open reading frames (ORFs; list in Supplementary data, Table S1 and Fig. 2a). The majority (62%) of predicted genes (57 ORFs) encoded hypothetical proteins with unknown functions, while 35 ORFs were homologous to functional proteins in the GenBank database in which have a high percentage (81–100%) identity to annotated proteins of phage in *Litunavirus* genus (Supplementary data, Table S2). The *Litunavirus* is a member of a well-characterized N4-like phage. Almost all of the N4-like phages exhibit highly conserved gene organization and expresion^21,22^. Therefore, the expressions of phage VL1 early, middle and late genes cluster are classified based on the N4-like phages as follows:

(i) **Early genes.** The putative early region of the VL1 genome encodes essential proteins involved in the transcription module, including putative transcriptional regulators (ORF6 and ORF18) and the N4 gp14-like protein (ORF17), which was recently demonstrated to function as an ssDNA-binding RNA polymerase cofactor^23^. Located further downstream, three genes encode RNA polymerase: RNA polymerase small subunit (ORF 21), putative RNAP2 (ORF 24), and RNA polymerase large subunit (ORF 26). The presence of the three RNA polymerase encoding genes suggests that phage VL1 has its own transcriptional machinery, functioning independently of the host RNA polymerase.
(ii) **Middle genes.** Most of the proteins encoded by middle genes with known functions are involved in DNA replication and nucleotide metabolism. Genes encoding these proteins are condensed in an area that tracts from ORF 38 to ORF 68. The proteins required for phage VL1 DNA replication include ATPase (ORF 38)**,** RecD-like DNA helicase YrrC (ORF 40)**,** putative DNA polymerase (ORF 42)**,** putative DNA primase P4 type (ORF 63), N4 gp44-like protein (ORF 64)**,** putative single-stranded DNA-binding protein (ORF 65), and N4 gp46-like protein (ORF 68), which are homologous to a putative RuvC-like Holliday junction resolvase. The presence of phage-encoded DNA polymerase, primase, and helicase indicated that phage VL1 elongates dsDNA independently of the host replication machinery. Genes encoding enzymes involved in DNA metabolism included putative dCMP deaminase (ORF 43), HNH endonuclease (ORF 46), and HNH endonuclease (ORF 51). Interestingly, phage VL1 carries a lysis inhibitor cassette, including rIIA-like protein (ORF 47) and rIIB-like protein (ORF 48), located between the DNA replication and nucleotide metabolism clusters. These proteins might play a role in the delay in host lysis resulting in a large increase in the phage burst size, providing phage VL1 with a competitive advantage over others.
(iii) **Late genes.** The majority of proteins encoded by late genes are involved in phage assembly for building the virion structure, packing proteins, and lysis of host cells. Proteins involved in virion morphology include tail fiber protein (ORF 56 and 59), lytic tail fiber (ORF 75 and 76), structural protein (ORF79 and ORF 87), major capsid protein (ORF 81), and putative portal protein (ORF 84). We predicted two proteins involved in DNA packing: the putative large terminase subunit (ORF 88), which is highly divergent from other phage sequences, and the N4 gp69-like protein (ORF 89), which has an appropriate size and genome location as a small terminase subunit. The VL1 genome also encodes a virion-associated RNA polymerase (ORF 74), a remarkable protein that is characteristic of N4-like viruses and unique among all other phages. N4-like viruses co-inject this enzyme with DNA during infection, and this virion-associated RNA polymerase is responsible for the transcription of early genes^24^. The lysozyme-encoding gene (ORF54) is also present in the VL1 genome; this protein is responsible for host cell lysis to release phage progeny from the host cell.

Remarkably, no genes encoding toxins, antimicrobial resistance-related functions, or genes encoding for lysogeny-associated proteins such as integrase, excisionase, recombinase, and repressor genes were identified, supporting the notion that phage VL1 is a virulent phage and can be used for therapeutic purposes.

### Phage vB_PaeS_VL1 is a novel *Pseudomonas* phage belonging to the *Litunavirus* genus

To determine the relationship between phage VL1 with other phages, Blastn (Megablast) comparison of VL1 genome sequence against the NCBI non-redundant database was performed. VL1 genome sequence was found to be similar (up to 97.04% nucleotide identity over 97% query coverage; see Supplement data, Table S3) to *Pseudomonas* phage genome sequences belonging to the genus *Litunavirus* subfamily *Migulavirinae,* family *Schitoviridae* and order *Caudovirales*. The family *Schitoviridae* is a new family of N4-like phages that was recently removed from the family *Podoviridae*^22^. To classify phage VL1 accurately, pairwise genome alignments were performed with members of the *Litunavirus* genus. The results revealed that VL1 has whole genome identity varied from 93.1% to 48.6% when compared to *Pseudomonas* phages YH6 (KM974184.1) and vB_PaeS_TUMS_P81 (OL519844.1), respectively (Supplementary data, Table S4).

Moreover, we used multiple genome alignments to compare the genome sequences of phage VL1 with homologous phages in the *Litunavirus* genus (Fig. 2b) and observed a considerable relationship between phage VL1 and other *Pseudomonas* phages within the *Litunavirus* genus. In correlation with the pairwise genomic alignment results, VL1 showed a substantial relationship with other *Pseudomonas* phages within the *Litunavirus* genus. However, similar regions were segmented between them. Taken together, the genome of phage VL1 differs by more than 5% from that of other *Pseudomonas* phages at the nucleotide level suggesting that it is a novel species belonging to this *Litunavirus* genus. This suggestion is in accordance with the guideline of novel species identification proposed by the International Committee on Taxonomy of Viruses (ICTV).

**Figure 3:**
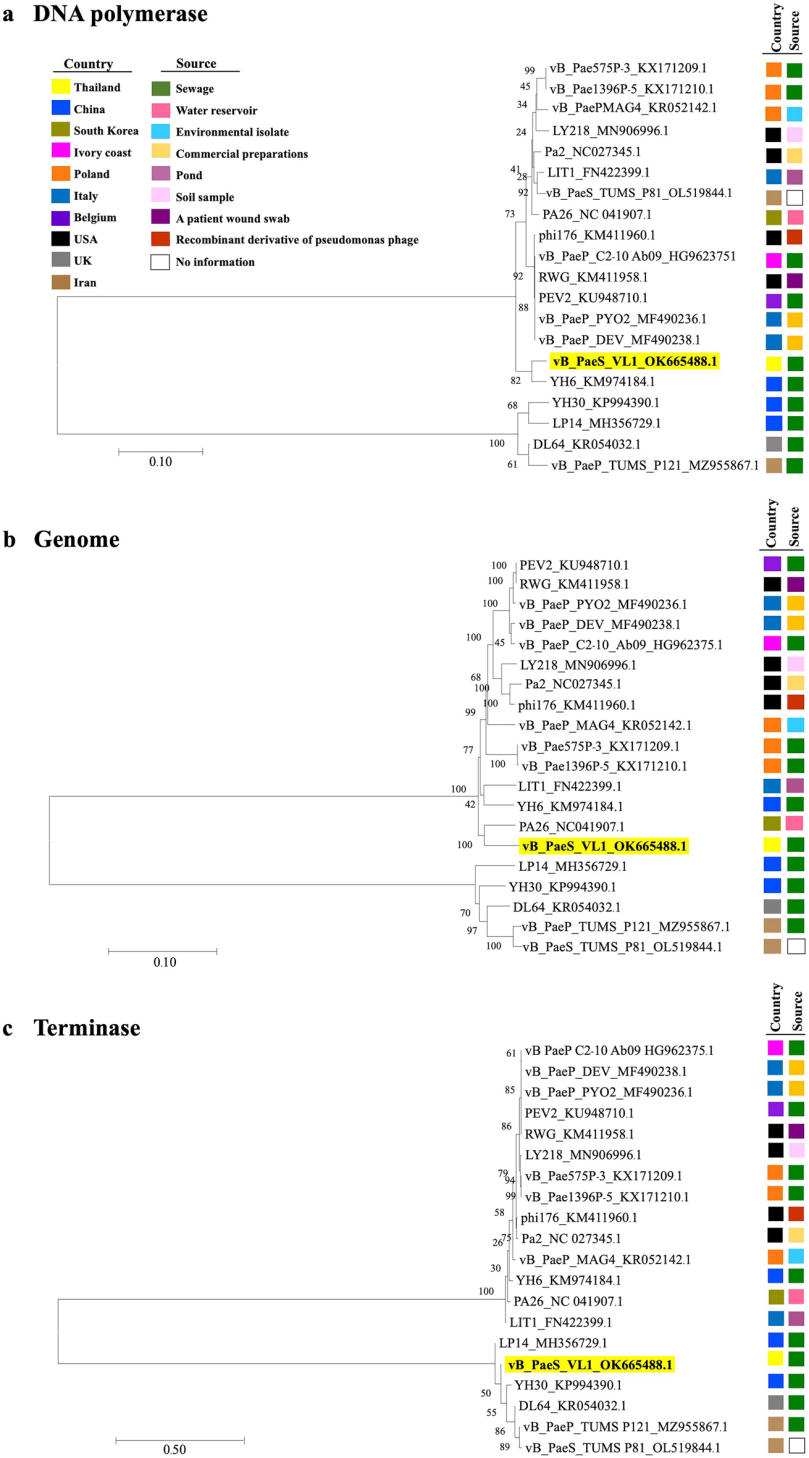
Neighbor-Joining phylogenetic tree based on (**a**) DNA polymerase gene, (**b**) complete genome sequences, or (**c**) terminase large subunit gene of phage vB_PaeS_VL1 and related phages in *Litunarviruses.* Numbers are shown next to the branches indicated by the percentage of replicate trees of the bootstrap test (1000 replicates). The evolutionary distances were processed using the Maximum Composite Likelihood method. The isolation source and country of each species are presented with a different colored rectangle.

To further investigate the exact taxonomic position of phage VL1, neighbor-joining (NJ) phylogenetic trees between newly isolated phage VL1 and 19 other phages belonging to the *Litunavirus* genus were constructed based on (a) DNA polymerase, (b) whole genome sequence, and c) terminase large subunit comparisons. As expected, the DNA polymerase gene of phage VL1 was most closely related to that of the *Pseudomonas* phage YH6 (KM974184.1), which was isolated from a sewage sample in China. Both genes showed a divergence of 0.036 base substitutions per site and high bootstrap values (Fig. 3a). However, the whole genome phylogenetic tree was partially inconsistent with the DNA polymerase-based phylogenetic tree, as phage VL1 was most closely related to *Pseudomonas* phage PA26 (NC041907.1), isolated from a water reservoir in South Korea, with a divergence of 0.064 base substitutions per site. This was followed by a more distant relationship with *Pseudomonas* phage YH6 and *Pseudomonas* phage LIT1 (FN422399.1) isolated from a pond in Italy, with divergences of 0.067 and 0.082 base substitutions per site, respectively (Fig. 3b).

The phylogenetic tree inferred using the terminase large subunit gene revealed that phage VL1 was affiliated with a branch formed by *Pseudomonas* phages YH30 (KP994390.1), DL64 (KR054032.1), vB_PaeP_TUM_P121 (MZ955867.1), and vB_PaeP_TUM_P81 (OL519844.1), with divergences of 0.023, 0.051, 0.051, and 0.053 base substitutions per site, respectively. All of these phages were isolated from sewage samples from different countries in Asia and Europe, that is, China, the United Kingdom, and Iran (Fig. 3c). The results of the phylogenetic analyses supported that phage VL1 is a new species within the *Litunavirus* genus.

### Phage adsorption rate, one-step growth curve and bacterial lysis efficiency

**Figure 4:**
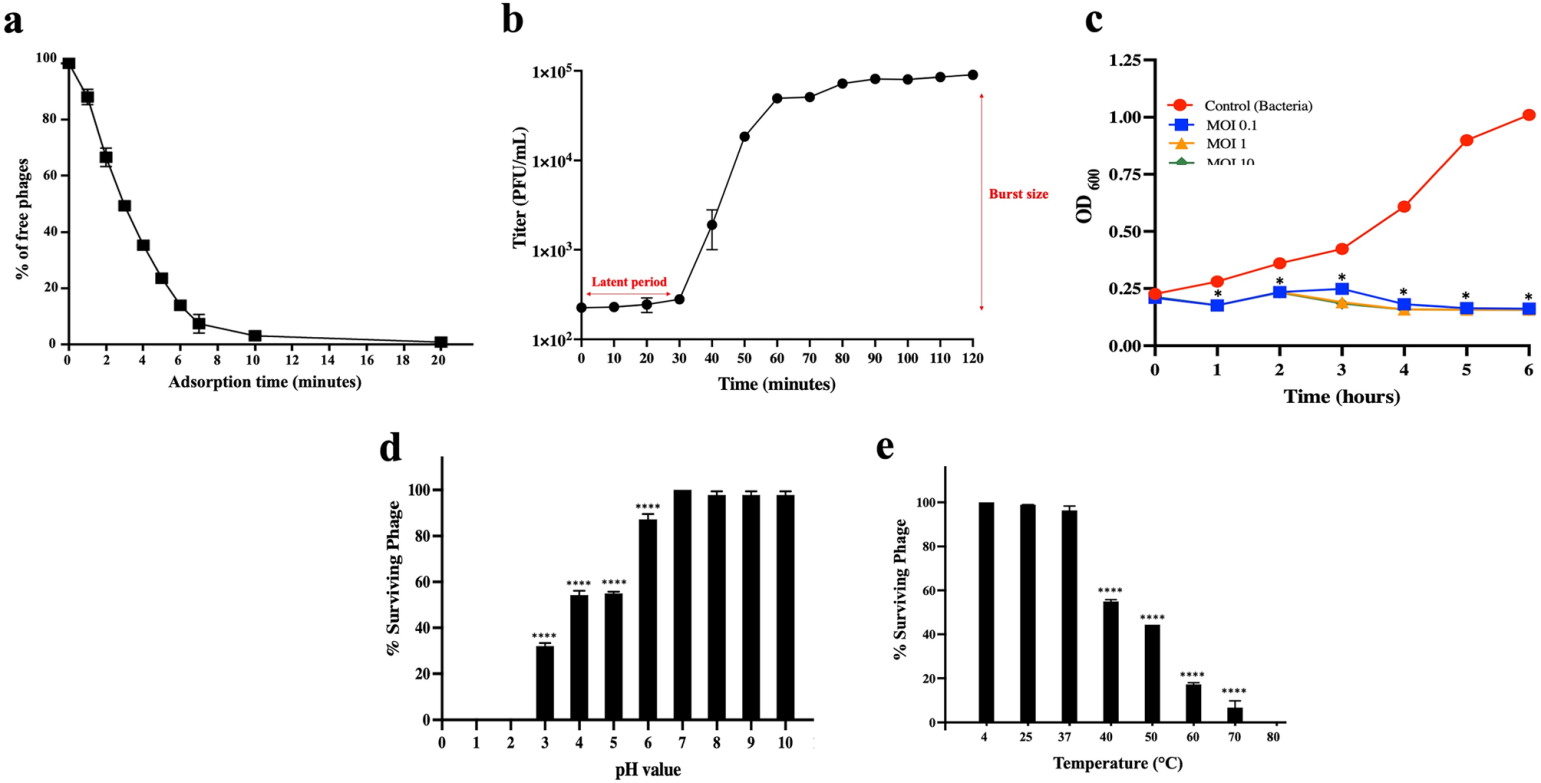
In vitro characterization of the phage vB_PaeS_VL1. (**a**) Kinetic adsorption of the phage to the host strain (*P. aeruginosa* ATCC27853) at an MOI of 0.01. (**b**) One-step growth curve of phage VL1 at a MOI of 0.001. (**c**) Bacterial killing activity of phage VL1 against host cells at different MOI (0.1, 1, and 10) in TSB medium. (**d**) Phage VL1 stability under different pH conditions. (**e**) Stability of Phage VL1 under different temperature conditions. Data are presented as mean ± SD of three independent experiments. The asterisks indicate significant differences (*P < 0.05 and ****P < 0.0001, one-way ANOVA followed by Dunnett's post hoc test) between the experimental and control groups.

The therapeutic potential of phage VL1 was initially characterized by infection parameters. To determine the adsorption rate of VL1 on the surface of *P. aeruginosa*, an adsorption assay was performed (Fig. 4a). The data revealed that ~ 50% of the phage rapidly attached to the *P. aeruginosa* ATCC 27853 within 3 min, and 90% of the phage subjected to the bacteria absorbed to them within 10 min. In addition, a one-step growth curve revealed that the latent period of phage VL1 was approximately 30 min, and the average burst size of one lytic cycle was 404 ± 10 plaque-forming units (PFU) per infected cell (Fig. 4b). These results indicated that phage VL1 exhibits a high rate of phage adsorption and progeny production which strengthens the therapeutic potential of this isolated phage.

We then further investigated the bacterial lysis activity of phage VL1 in broth culture medium. *P. aeruginosa* ATCC27853 was infected with phage VL1 at various multiplicities of infection (MOIs) of phage (0.1, 1, and 10). As shown in Fig. 4c, the optical density (OD_600_) values of *P. aeruginosa* incubated with phage VL1 at MOI of 0.1, 1, and 10 were significantly lower than the control (*P* < 0.05) at all time points, including 1, 2, 3, 4, 5 and 6 h after incubation. The decrease in optical density indicates the lysis of bacterial cells. In contrast, *P. aeruginosa* without phage treatment (control) showed increased bacterial growth during 6 h of incubation. Remarkably, there was no significant difference (*P* > 0.05) in bacterial growth when different MOI (0.1, 1, and 10) were applied for phage treatment, indicating that even a low MOI (0.1) is enough to inhibit bacterial regrowth (Fig. 4c). Taken together, these data suggested that phage VL1 could effectively inhibit the growth of *P. aeruginosa* with a broad range of MOI infection (0.1–10).

### pH and thermal stability of phage vB_PaeS_VL1

Because the high stability of phage over a wide range of pH and temperatures is advantageous for potential phage therapy applications, VL1 was characterized in terms of its pH and thermal stability. As shown in Fig. 4d, the titer of phage VL1 remained almost unchanged following incubation at pH values ranging from 7 to 10. However, the phage titer was significantly reduced in the samples incubated at a pH of 3 to 6 when compared to the pH of 7 (*P* < 0.0001). Phage VL1 was completely inactivated when exposed to strongly acidic pH values of 2 and 1. Additionally, as compared to storage conditions (4 °C), phage VL1 was substantially stable at 25 °C and 37 °C. However, the phage VL1 activity considerably diminished (*P* < 0.0001) when exposed to temperatures rising from 40 °C to 70 °C, and it was entirely inactivated at 80 °C after an hour of incubation (Fig. 4e).

### Killing efficiency of phage vB_PaeS_VL1 against clinical isolates of *P. aeruginosa*

We further investigated the lytic activity of phage VL1 against 60 clinical isolates of *P. aeruginosa* collected from companion animal diseases such as skin and soft tissue infection as well as urinary tract infection (listed in Table 1). All of them are MDR isolates. The results showed that VL1 can infect and form clear plaques in 34 of 60 tested strains (56%) (Table 1). Moreover, no lytic activity against other Gram-negative and Gram-positive bacteria, including *Escherichia coli*, *Burkholderia thailandensis*, *Proteus mirabilis*, *Staphylococcus aureus*, *Enterococcus faecalis* was observed, indicating the high specificity of phage VL1 infection.

**Table 1:** Host range infection and EOP of phage vB_PaeS_VL1 against 61 *Pseudomonas* and other bacteria. The lytic activity was determined by plaque assay. The results “ + ” indicates lytic or susceptibility; “ − ” indicates non − lytic or resistant; ND = Not determine.

Strains	Drug Resistance	Source	Lytic activity	Efficiency of plating
*P. aeruginosa* ATCC27853	AMP AMC CL FOX CPD SXT	Reference strain	+	1.00 ± 0.00 (Propagation host)
PA898	AMP AMC CL FOX CRO CPD CAZ CTX ENR SXT TE DO OT AK CN	Ear swab/Cat	+	0.61 ± 0.01 (High)
PA900	AMP AMC CL FOX CRO CPD SXT TE DO OT	Nasal swab/Cat	−	ND
PA910	AMP AMC CL FOX CRO CPD CTX SXT TE DO OT	Wound swab/Dog	+	0.08 ± 0.01 (Low)
PA947	AMP AMC CL FOX CRO CPD NOR CIP ENR SXT TE DO OT CN	Wound swab/Dog	+	0.75 ± 0.04 (High)
PA881	AMP AMC CL FOX CRO CPD CTX SXT TE DO OT	Wound swab/Dog	+	0.08 ± 0.02 (Low)
PA1005	AMP AMC CL FOX CRO CPD CTX NOR CIP ENR SXT TE DO OT CN	Wound swab/Cat	−	ND
PA1083	AMP AMC CL FOX CRO CPD CTX ENR SXT TE DO OT	Wound swab/Dog	+	0.05 ± 0.02 (Low)
PA1093	AMP AMC CL FOX CRO CPD CTX SXT TE DO OT	Abdominal/Dog	+	0.88 ± 0.05 (High)
PA1017–61	AMP AMC CL FOX CRO CPD CTX ENR SXT TE DO OT	Wound swab/Cat	+	0.85 ± 0.01 (High)
PA1170	AMP AMC CL FOX CRO CPD CTX SXT TE DO OT	Urine/Cat	−	ND
PA1171	AMP AMC CL FOX CRO CPD CTX SXT TE DO OT	Wound swab/Dog	+	0.85 ± 0.05 (High)
PA729	AMP AMC CL FOX CPD CTX SXT TE DO OT	Ear swab/Cat	+	0.77 ± 0.01 (High)
PA708	AMP AMC CL FOX CRO CPD CAZ CTX ENR SXT TE DO OT AK CN	Ear swab/Dog	−	ND
PA819	AMP AMC CL FOX CRO CPD CTX ENR SXT TE DO OT	Urine/Cat	+	0.84 ± 0.02 (High)
PA659–2	AMP AMC CL FOX CRO CPD CTX SXT TE DO OT	Eye swab/Dog	+	0.04 ± 0.01 (Low)
PA700	AMP AMC CL FOX CPD CTX SXT TE DO OT	Wound swab/Dog	−	ND
PA814	AMP AMC CL FOX CRO CPD CAZ CTX ENR SXT TE DO OT	Wound swab/Dog	−	ND
PA801	AMP AMC CL FOX CRO CPD CTX ENR SXT TE DO OT CN	Wound swab/Dog	+	0.68 ± 0.01 (High)
PA728	AMP AMC CL FOX CRO CPD CAZ CTX ENR SXT TE DO OT	Urine/Cat	+	0.74 ± 0.03 (High)
PA722	AMP AMC CL FOX CRO CPD CAZ CTX ENR SXT TE DO OT	Urine/Dog	+	0.79 ± 0.01 (High)
PA791	AMP AMC CL FOX CRO CPD CAZ CTX SXT TE DO OT	Wound swab/Cat	−	ND
PA729	AMP AMC CL FOX CPD CTX SXT TE DO OT	Ear sawb/Dog	+	0.81 ± 0.03 (High)
PA723–2	AMP AMC CL FOX CRO CPD CTX SXT TE DO OT	Urine/Dog	−	ND
PA755–2	AMP AMC CL FOX CPD CTX SXT DO OT	Nasal swab/Cat	−	ND
PA392	AMP AMC CL FOX CPD CTX SXT TE DO	Nasal swab/Cat	+	0.78 ± 0.05 (High)
PA34	AMP AMC CL FOX CRO CPD CAZ CTX NOR CIP ENR SXT TE DO OT CN	Urine/Dog	−	ND
PA294	AMP AMC CL FOX CRO CPD CTX NOR CIP ENR SXT TE DO OT AK CN	Thoracic cavity/Cat	+	0.45 ± 0.02 (Moderate)
PA149–4	AMP AMC CL FOX CRO CPD CAZ CTX NOR CIP ENR SXT TE DO OT CN	Wound swab/Dog	−	ND
PA897	AMP AMC CL FOX CRO CPD CTX SXT TE DO OT AK CN	Wound swab/Dog	+	0.89 ± 0.03 (High)
PA41	AMP AMC CL FOX CRO CPD CTX NOR CIP ENR SXT TE DO OT AK CN	Urine/Dog	−	ND
PA269	AMP AMC CL FOX CRO CPD CAZ CTX NOR CIP ENR SXT TE DO OT AK CN	Urine/Dog	+	0.05 ± 0.01 (Low)
PA276	AMP AMC CL FOX CRO CPD CTX ENR SXT TE DO OT CN	Urine/Dog	−	ND
PA212	AMP AMC CL FOX CRO CPD CAZ CTX NOR SXT TE DO OT	Ear swab/Dog	−	ND
PA422	AMP AMC CL FOX CRO CPD CTX ENR SXT TE DO OT	Wound swab/Dog	−	ND
PA261	AMP AMC CL FOX CRO CPD CTX ENR SXT TE DO OT	Wound swab/Cat	+	0.85 ± 0.03 (High)
PA280	AMP AMC CL FOX CRO CPD CTX ENR SXT TE DO OT	Wound swab/Dog	−	ND
PA187–2	AMP AMC CL FOX CRO CPD CAZ CTX SXT TE DO	Wound swab/Dog	+	0.77 ± 0.02 (High)
PA278	AMP AMC CL FOX CRO CPD CAZ CTX SXT TE DO OT	Wound swab/Cat	−	ND
PA371–2	AMP AMC CL FOX CRO CPD CAZ CTX NOR CIP ENR SXT TE DO OT AK CN	Wound swab/Dog	−	ND
PA1033	AMP AMC CL FOX CRO CPD CTX SXT TE DO OT	Urine/Dog	+	0.88 ± 0.02 (High)
PA1092	AMP AMC CL FOX CPD MAR CTX SXT TE DO OT	Ear swab/Dog	+	0.76 ± 0.04 (High)
PA1122	AMP AMC CL FOX CRO CPD CTX NOR CIP ENR MAR SXT TE DO OT	Urine/Dog	+	0.85 ± 0.02 (High)
PA1017–64	AMP AMC CL FOX CPD SXT TE DO	Urine/Dog	+	0.75 ± 0.05 (High)
PA1147	AMP AMC CL FOX CPD CTX SXT TE DO OT	Urine/Cat	−	ND
PA1144	AMP AMC CL FOX CRO CPD CTX SXT TE DO OT	Urine/Cat	−	ND
PA1030	AMP AMC CL FOX CPD CTX NOR SXT TE DO OT	Ear swab/Dog	−	ND
PA970	AMP AMC CL FOX CRO CPD CTX SXT TE DO OT	Urine/Dog	+	0.87 ± 0.03 (High)
PA1069	AMP AMC CL FOX CRO CPD CTX NOR CIP ENR MAR SXT TE DO OT CN	Wound swab/Dog	−	ND
PA703	AMP AMC CL FOX CRO CPD CAZ ENR CTX SXT TE	Pus/Dog	+	0.91 ± 0.03 (High)
PA720	AMP AMC CL FOX CRO CPD CTX SXT TE DO OT	Urine/Cat	+	
PA286	AMP AMC CL FOX CRO CPD CTX SXT TE DO OT	Wound swab/Dog	−	ND
PA284	AMP AMC CL FOX CRO CPD CTX SXT TE DO OT AK CN	Urine/Dog	+	0.37 ± 0.02 (Moderate)
PA270	AMP AMC CL FOX CRO CPD CAZ CTX ENR SXT TE DO OT	Pus/Dog	+	0.35 ± 0.06 (Moderate)
PA12	AMP AMC CL FOX CRO CPD CTX SXT TE DO OT	Wound swab/Cat	+	0.85 ± 0.01 (High)
PA15	AMP AMC CL FOX CRO CPD NOR CIP ENR CTX SXT TE DO OT AK CN	Ear swab/ Cat	−	ND
PA53	AMP AMC CL FOX CRO CPD CAZ ENR SXT TE DO OT	Abdominal fluid/ Cat	+	0.61 ± 0.02 (High)
PA80	AMP AMC CL FOX CRO CPD CTX ENR SXT TE DO OT	Wound swab/Dog	−	ND
PA89	AMP AMC CL FOX CRO CPD CAZ NOR CIP CTX SXT TE DO OT	Wound swab/Dog	+	0.33 ± 0.02 (Moderate)
PA108	AMP AMC CL FOX CRO CPD CTX ENR SXT TE DO OT	Wound swab/Cat	−	ND
PA112	AMP AMC CL FOX CRO CPD NOR CIP ENR CTX SXT TE DO OT	Wound swab/Cat	+	0.007 ± 0.01 (Low)
*Escherichia coli* ATCC25922	ND	Reference strain	−	ND
*Burkholderia thailandensis* E264	ND	Environmental sample	−	ND
*Proteus mirabilis* 22–65	AMP SXT TE DO OT	Wound swab/Dog	−	ND
*Staphylococcus aureus* ATCC25923	ND	Reference strain	−	ND
*Enterococcus faecalis* ATCC29212	ND	Reference strain	−	ND

To semi-quantitate, the lytic activity of phage VL1, an efficiency of plating (EOP) assay was performed on 34 MDR *P. aeruginosa* strains that were susceptible to phage VL1. High, moderate and low productive infection of phage VL1 was found for 24, 4, and 6 isolates, respectively (Table 1) suggesting that the majority of susceptible MDR *P. aeruginosa* (approximately 70.6%; 24/34) could be lysed by phage VL1 with high efficiency. These data supported the potential of phage VL1 to be used as an anti-*P. aeruginosa* agent in veterinary medicine.

**Figure 5:**
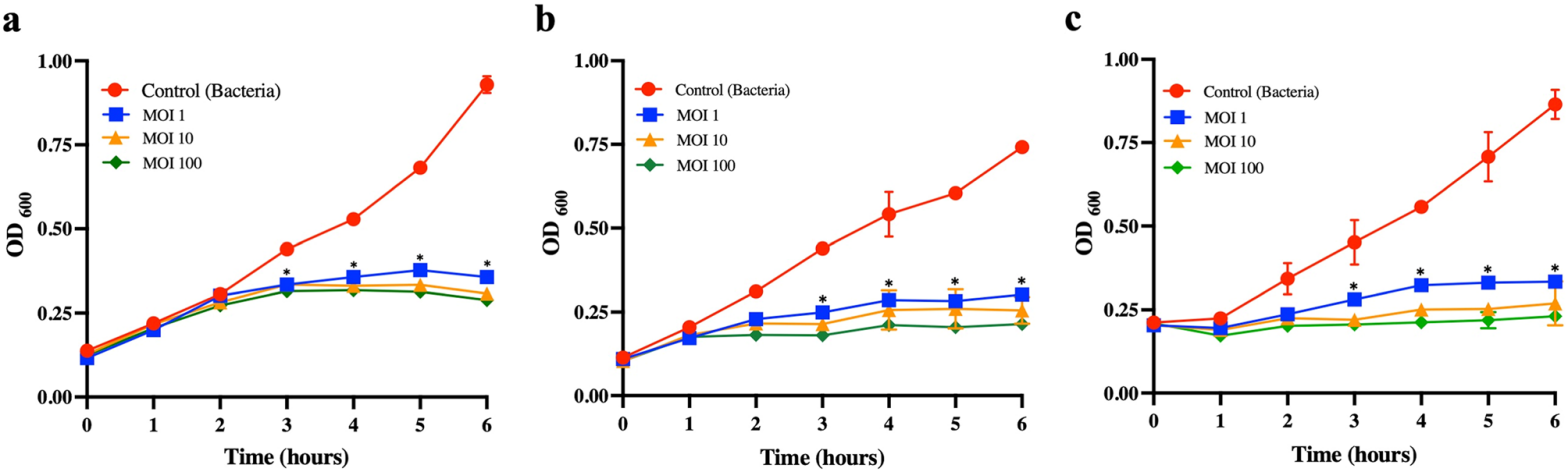
Efficiency of the lytic activity of the phage vB_PaeS_VL1 against three different clinical isolates of MDR *P. aeruginosa.* (**a**) The *P. aeruginosa* strains MDR PA897, (**b**) MDR PA269 or (**c**) MDR PA294 were cocultured with the phage vB_PaeS_VL1 at different MOIs (i.e., MOI of 1, 10 and 100) and incubated at 37 °C. Every 1 h for 6 h, bacterial growth was measured by optical density measurement. Bacterial culture without phages was included as a control. Data are presented as mean ± SD of three independent experiments. The asterisks indicate significant differences (*P < 0.05, one-way ANOVA followed by Dunnett's post hoc test) between the experimental and control groups.

The ratio of phage to target bacteria should affect the efficiency of bacterial lysis. Next, we further investigate the various amounts of VL1 phage incubated with the target bacteria. In this case, the target bacteria include 3 different strains of MDR *P. aeruginosa*, i.e., strains PA897 and PA269, isolated from soft tissue wound swabs and urine samples from dogs, respectively, while strain PA294 was isolated from the thoracic cavity of a cat. The bacteria (10^8^ CFU/mL) were cocultured with phage VL1 in a liquid medium with three different MOI (1, 10 and 100). As shown in Fig. 5a–c, the growths of the MDR *P. aeruginosa* strains PA897, PA269 and PA264 treated with phage VL1 at MOI of 1, 10 or 100 were significantly reduced after 3, 4, 5 and 6 h of incubation compared to those of the control groups (*P* < 0.05). Furthermore, there was no significant difference (*P* > 0.05) in the bacterial density when varying MOI (1, 10 and 100), at all time points studied (Fig. 5a–c). On the contrary, the growth of the three MDR *P. aeruginosa* strains without phage VL1 treatment (control) increased continuously over time from the beginning to 6 h of incubation. This result indicated that phage VL1 could efficiently suppress the growth of MDR *P. aeruginosa* from 3 to 6 h in all MOI (1–100), suggesting that phage VL1 has a broad range of effective therapeutic amounts which could be beneficial when applied to patients.

### Biofilm eradication efficacy against clinical isolated MDR *P. aeruginosa*

**Figure 6:**
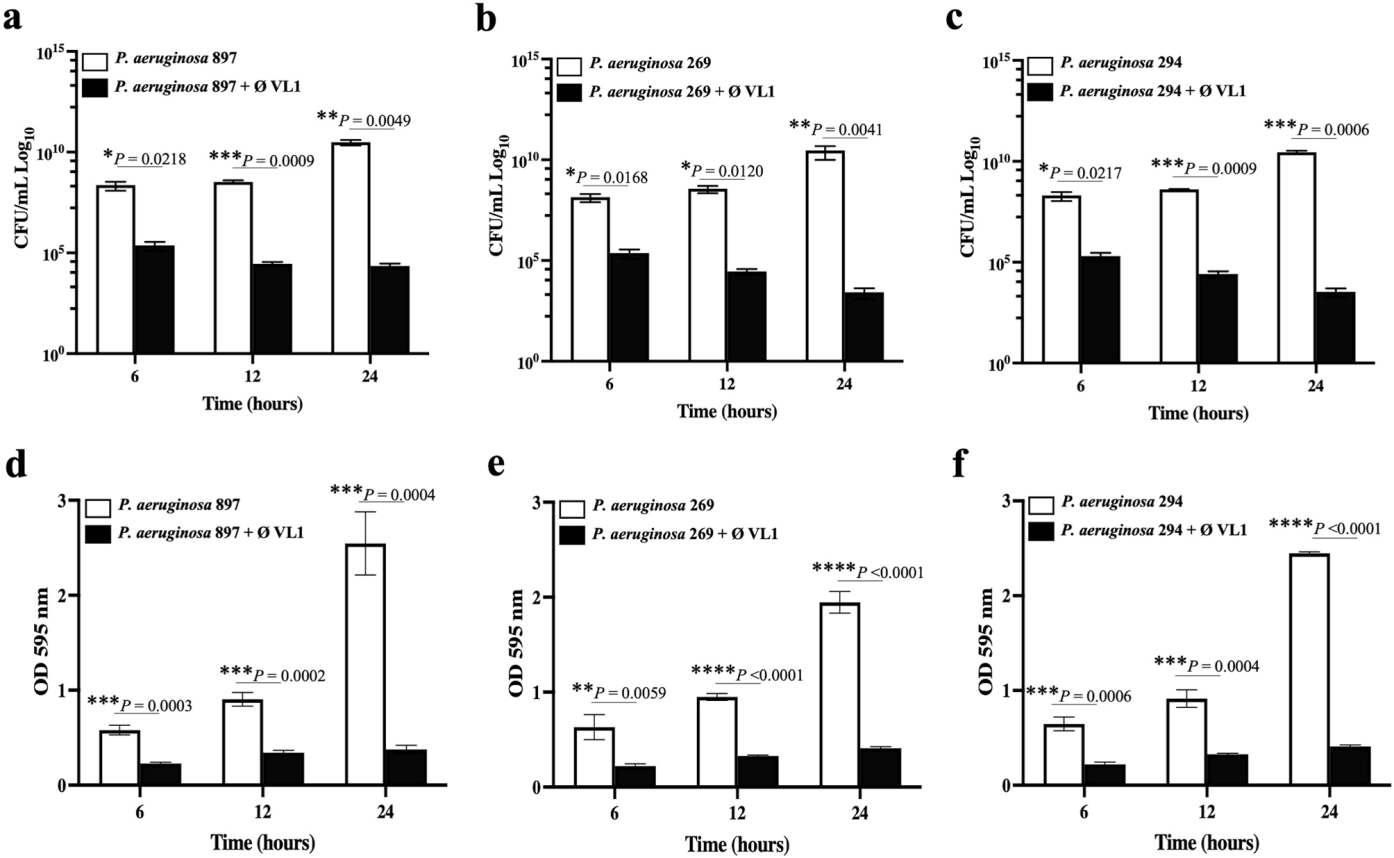
Biofilm eradication activity of the phage vB_PaeS_VL1 against clinical isolates of MDR *P. aeruginosa.* The viable bacterial cell counts in 48 h-old biofilms of *P. aeruginosa* strains (**a**) MDR PA897, (**b**) MDR PA269 or (**c**) MDR PA294 after treatment with phage VL1 for 6, 12 and 24 h were measured using the standard plate count. The total biofilm mass of 48 h-old biofilms of *P. aeruginosa* strains (**d**) MDR PA897, (**e**) MDR PA269 or (**f**) MDR PA294 after treatment with phage VL1 for 6, 12 and 24 h. The absorbance of each well was measured at 595 nm after a crystal violet stain. Data are presented as mean ± SD of three independent experiments. ****P < 0.0001, ***P < 0.001, **P < 0.01 or *P < 0.05, Students *t* test, indicates statistically significant differences between experimental and control groups.

The ability of *P. aeruginosa* to form biofilm is a major problem that leads to treatment failure due to antibiotic resistance^25^. Next, we investigated the therapeutic potential of phage VL1 to eradicate the MDR *P. aeruginosa* biofilm*.* MDR *P. aeruginosa* strains PA897, PA269 and PA294 were attached to a polystyrene plate for 48 h, leading to the establishment of a mature biofilm before adding the phage VL1. The VL1 phage was shown to have a strong ability to destroy the existing biofilm produced by the MDR *P. aeruginosa* strains PA897, PA269 and PA294 (Fig. 6a–f). During the first 6 h of phage VL1 treatment, the number of viable PA897, PA269, or PA294 in the biofilm was significantly lower than in the control without phage treatment (*P* < 0.05). The numbers (CFU/mL) of PA897, PA269 and PA294 in the biofilm decreased by approximately 3 log reduction (*P* < 0.05) at 6 h of treatment and 4 log reduction (*P* < 0.05), 6 log reduction (*P* < 0.01) at 12 and 24 h, respectively (Fig. 5a–c), indicating that phage VL1 effectively removed the biofilm in a time-dependent manner.

In addition to counting the number of viable bacteria after phage treatment, a measurement of biofilm mass was performed by crystal violet staining. At 6 h after phage treatment, a significant reduction in biofilm mass was observed (57–64% reduction; *P* < 0.01), generated from the three MDR *P. aeruginosa* strains, compared to the untreated control (Fig. 6d–f). At the end of the experiment (24 h) the reduction was even greater, reaching an average biomass reduction of 75% (*P* < 0.001), confirming the effective antibiofilm activity of phage VL1. Notably, we could not observe the biofilm reduction before 6 h of phage treatment.

### Therapeutic efficacy against clinical MDR *P. aeruginosa* in *G. mellonella* model

**Figure 7:**
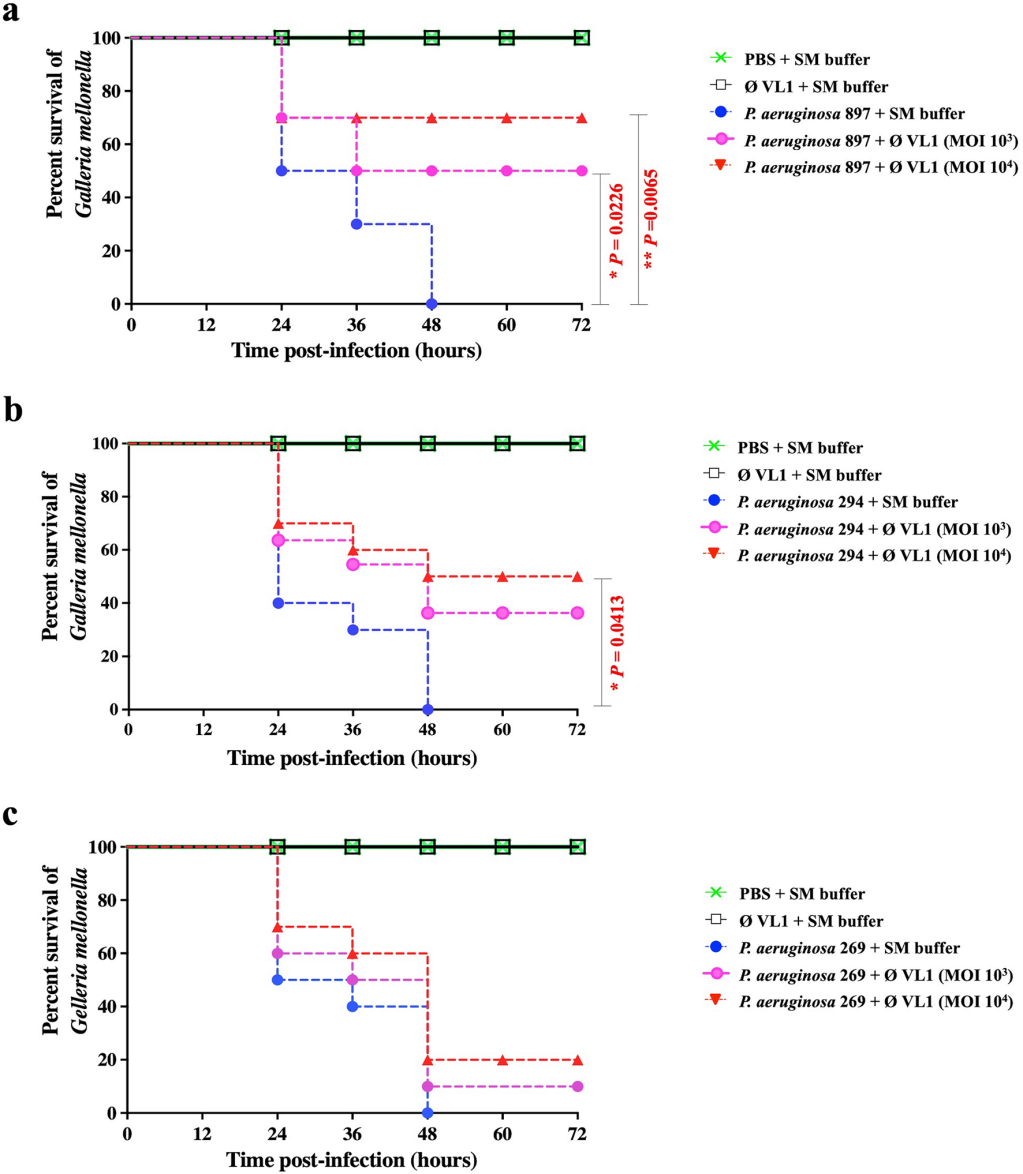
Percent survival of MDR *P. aeruginosa* infected *G. mellonella* larvae after phage vB_PaeS_VL1 treatment. Groups of 10 larvae were inoculated with either MDR *P. aeruginosa* strains (**a**) PA897, (**b**) PA294 or (**c**) PA269 follow by treatment with phage VL1 at MOI of 1,000 or 10,000 at 1 h p.i.. The numbers of dead larvae were determined every 12 h for 72 h p.i. GraphPad Prism software was used to graph and analyze the data using a Log-rank (Mantel-Cox) test. Asterisks indicated significant differences (*P < 0.05 or **P < 0.01) between bacterial infection groups, and the phage treatment groups. Data is representative of that obtained in three independent experiments (n = 3 biological replicates).

*G. mellonella* larvae were used as an animal infection model to assess the in vivo therapeutic efficacy of phage VL1 against MDR *P. aeruginosa* clinical strains (PA897, PA294 and PA269)*.* The results demonstrated that all *G. mellonella* larvae receiving only PBS or phage lysate showed a 100% survival rate up to 72 h, implying VL1 was not toxic for larvae. In contrast, all larvae infected with strains MDR PA897, PA294 or PA269 but not treated with phage VL1 died within a period of 48 h (Fig. 7a–c).

Treatments of phage VL1 against larvae infected with *P. aeruginosa* strains were assessed under two different phage doses (MOI of 10^3^ and 10^4^). At MOI 10^3^, phage VL1 significantly improved the survival rate to 50% (*P* = 0.0226) in MDR PA897-infected larvae at the end of observation period (72 h) (Fig. 7a). In contrast, there was no significant difference in the survival rate of MDR PA294-infected larvae after phage VL1 treatment at MOI 10^3^ (*P* > 0.05) (Fig. 7b,c). When MOI was increased to 10^4^, phage VL1 significantly enhanced the survival rate of larvae infected with *P. aeruginosa* strains MDR PA897 and MDR PA294 up to 70% (*P* = 0.0065) (Fig. 7a) and 50% (*P* = 0.0413) (Fig. 7b), respectively. Nevertheless, there was no significant difference (*P* > 0.05) in the survival rate of MDR PA269-infected larvae treated with phage VL1, only 10% of the larvae were rescued (Fig. 7c).

## Discussion

*P. aeruginosa* is one of six bacterial pathogens in the ESKAPE group and is categorized by the Centre for Disease Control and Prevention as a serious threat due to the development of multidrug resistance and the increasing number of hospitalizations^26^. This is not only a concern in human health but also animal health, as noticed by the increase in antibiotic-resistant bacterial infections among companion animals^27^. In the last decade, several researchers have focused their efforts on the isolation, characterization, and application of lytic phages against *P. aeruginosa* infection^28–30^. Here, we isolated and characterized the VL1, a novel *Pseudomonas* phage, as an alternative approach for MDR *P. aeruginosa* treatment.

Phage VL1 was successfully isolated from an urban sewage water sample in Thailand. This is correlated with a previous study that sewage-contaminated environments are the most prominent sources of novel phages^31^. The evolutionary relationship between the VL1 phage and previously reported *Litunarvirus* phages was evaluated by phylogenetic tree analysis of the entire genome and specific genes. Phage VL1 is novelty and closely related to phage isolated from Asian countries, as shown in Fig. 3a–c; these included phage PA26, isolated in Korea (NC_041907.1), phage YH6 (KM974184.1) and phage YH30 (KP994390.1) isolated in China but distant from phages isolated from western countries such as phage vB_Pae575P-3 (KX171209.1) isolated in Poland, phage PEV2 (KU948710.1) isolated in Belgium, and phage vB_Pae575P-3 (KX171209.1) isolated in Poland. This suggests that *Litunarvirus* phages have a wide geographic distribution. Since the geographic distribution depends on the abundance of the host and the metabolic state of the host^32^, more studies are required to identify the possible reason for this geographic distribution.

Through the predicted gene products, VL1 is a virulent phage and does not harbor any harmful genes, such as genes that encode toxins, antimicrobial resistance, making it a potentially attractive phage as a therapeutic agent. In contrast to lysogenic phages, which are not recommended for therapy^33^. Furthermore, as the VL1 phage has a short exact direct repeat end type of terminus, it is less prone to generalized transducing resistance genes than phages characterized as headful packaging^34^. Phage-mediated gene transfer should be avoided for phage therapy because it can also transduce harmful genes. Hence, genome characters indicate the potential of VL1 as a candidate for therapeutic value against *P. aeruginosa* infections. However, a large portion (61.95%) of the VL1 genome represented hypothetical proteins. This is a common problem in phage studies because functional identification of phage genes in microbial proteins^35^.

For biological properties, the plaque size of VL1 is markedly higher than closely related *Litunarvirus* namely YH6 (1–2 mm)^36^ and LP14 (2–3 mm)^37^. This may suggest that phage VL1 is smaller than the other 2 phages in the *Litunarvirus* family, as in general larger plaques often correlate with smaller phage head size. Data on phage adsorption and its growth parameters indicated that VL1 could be rapidly absorbed into host cells, with a short latent period and high average burst size. For comparison to the most closely related phage YH6 (Fig. 3a), although phage VL1 showed genome sequence similarities with YH6 (92%), their biological characteristics are different e.g. VL1 had burst size (around 404 ± 10 phages per cell) higher than phage YH6 (around 100 phages per cell)^36^. A large burst size could increase phage efficiency in phage therapy and reduce the potential for the emergence of phage-resistant bacteria.

Phages that have high stability at various temperatures and pH values could be selected as a good candidate for application as sanitizers or alternative therapeutic agents. Phage VL1 was able to survive, although with low titer, at pH 3–5. This character was similar to many phages in earlier studies, including the *Pseudomonas* phage LP14^37^. Furthermore, phage VL1 retained its activity at 37 °C, which is an average normal body temperature of humans, canines and felines, supporting the potential for therapeutic application in humans and companion animals. Although VL1 phage could infect 56% (34/60) of clinical MDR *P. aeruginosa* strains, it is higher than previously reported phages in *Litunarvirus;* e.g., phage LP14 showed 41% (7/17) lysis against *P. aeruginosa* strains^37^. However, a report claimed that *Pseudomonas* phage could cause lysis of up to 80% of *P. aeruginosa* isolates^36^. Interestingly, phage VL1 possessed strong lytic activity against three strains of MDR *P. aeruginosa* clinical isolates at low MOI, which is preferred because it could generate a lower immune response when applied in human or companion animal.

*P. aeruginosa* is known for its ability to form biofilms, and its tendency to accumulate bacteria in the early stages of infection^38^. Biofilms are difficult to eliminate by the host's immune defense and antimicrobial agents, and this makes *P. aeruginosa* infection more severe^39^. Previous studies have demonstrated that *Pseudomonas* phages possess antibiofilm activity^40–42^. In fact, phage VL1 was clearly evident to eradicate preformed biofilms from MDR *P. aeruginosa* strains, highlighting the possibility of using phage VL1 as a biocontrol agent. However, since our experiment was performed on a polystyrene plate, further research will focus on the other model, including a glass or a stainless-steel device to mimic the colonization of *P. aeruginosa* in hospital environments. Furthermore, biofilms generated in the laboratory are different from in vivo biofilms due to the presence of body fluid and host cells^43^.

*G. mellonella* was used as an in vivo model to evaluate the therapeutic efficacy of phage VL1. Our finding indicates that phage VL1 has therapeutic efficacy and suggests that it might be used as a candidate for a therapeutic agent. The phage VL1 appears to improve the survival rate of *G. mellonella* against clinical isolates of MDR *P. aeruginosa.* The highest increase in survival rate by 70% was observed in infected larvae treated with phage VL1 at MOI of 10^4^ for a period of 72 h. Moreover, the *G. mellonella* used in this study exhibited no toxicity from the concentrated phage injections. Antoine et al^44^ reported that a single dose of *Pseudomonas* phage at MOI of 5 × 10^4^ showed unsuccessful on therapy in larvae infected with *P. aeruginosa* dog otitis isolate. Another study reported that phages SL1, SL2 and SL4 treatments (MOI of 10 and 10^3^) improved the survival rate of MDR *P. aeruginosa* infected larvae up to 90% at 24 h post infection^40^.

In summary, vB_PaeS_VL1 is a novel isolated virulent phage, representative of the *Litunavirus* genus. The efficiency of anti-microbial activity and its ability to disrupt the biofilm and improve the survival of *G. mellonella* infected with clinical isolates of MDR *P. aeruginosa* indicated that it should be a candidate for therapeutic phage.

## Materials and methods

### Ethics statement

This study was reviewed and approved by the Institutional Biosafety Committee-IBC, Faculty of Veterinary Science, Mahidol University. The approval certificate number is IBC/MUVS-B-003/2564. All animal experiments followed the regulations of the Faculty of Veterinary Science, Mahidol University-institute animal care and use committee (FVS-MU-IACUC approval no. MUVS-2022–05-37).

### Bacterial strains and growth conditions

The 66 bacterial strains used in the present study are listed in Table 1. The sixty *P. aeruginosa* strains were isolated from cat or dog clinical specimens as part of routine service at the Microbiology Laboratory, Center of Veterinary Diagnosis, Faculty of Veterinary Science, Mahidol University. They were cultured in tryptone soy broth (TSB; HiMedia, India) or plated on a solid TSB medium (HiMedia). All four Gram-negative strains were cultured in TSB broth or agar. All two Gram-positive strains were cultured in brain heart infusion broth (BHI; HiMedia) or agar plates. The liquid cultures were grown with aeration at 37 °C in a shaking incubator, and the plates with the solid medium were incubated at 37 °C for 24 h.

### Antibiotic susceptibility profile of *P. aeruginosa*

Eighteen antibiotics were used to determine the antibiotic susceptibility profile of the *P. aeruginosa* clinical isolates using the disk diffusion method on Muller Hinton agar (HiMedia) according to the Clinical and Laboratory Standards Institute (CLSI) guidelines^45^. The following antibiotics (Oxoid™, UK or Mast group, UK) were included: 10 μg Ampicillin (AMP), 20/10 μg Amoxicillin/Clavulanic acid (AMC), 30 μg Ceftriaxone (CRO), 30 μg Cephalexin (CL), 30 μg Cefoxitin (FOX), 10 μg Cefpodoxime (CPD), 30 μg Ceftazidime (CAZ), 30 μg Cefotaxime (CTX), 5 μg Ciprofloxacin (CIP), 10 μg Norfloxacin (NOR), 5 μg Enrofloxacin Baytril (ENR), 25 μg Trimethoprim/ sulfamethoxazole (SXT), 30 μg Tetracycline (TE), 30 μg Doxycycline (DO), 30 μg Oxytetracycline (OT), 30 μg Amikacin (AK), 10 μg Gentamicin (CN) and 5 μg Marbofloxacin (MAR). The reference strain, *P. aeruginosa* ATCC27853, was used as a control. Multidrug-resistant strains were defined as those resistant to three or more classes of antibiotics.

### Phage isolation and purification

Phages were isolated from sewage samples using standard spot and double-layer plaque assays, as previously described with some modifications^17^. In brief, 20 mL of sewage water was filtrated through 0.45 µM pore-size membrane filters (Millipore, USA) and then mixed with 20 mL of double-concentrated TSB medium (HiMedia, India). The exponential growth phase of *P. aeruginosa* ATCC27853 (a host for phage isolation) was added to the mixture and incubated at 37 °C with shaking for 24 h. Thereafter, the mixture was centrifuged and filtered through 0.45 µM membrane filters. To screen for lytic phages, the supernatant was spotted on the *P. aeruginosa* ATCC27853 lawn. The clearance of the lawn in the spot was confirmed using a double-layer plaque assays^46^. To purify the isolated phage, a single plaque was plaque-purified at least five times.

### Microscopic analysis

To observe phage morphology by transmission electron microscopy (TEM), phages were fixed with 2.5% glutaraldehyde in 0.1 M phosphate buffer (pH 7.3) for 3 h. A drop of this solution was dropped into a Formvar-carbon-coated copper grid (200 mesh) and negatively stained with 2% uranyl acetate. The stained specimens were dried on Whatman No.1 filter paper and observed under an FEI Tecnai G2 T20 transmission electron microscope with an acceleration voltage of 120 kV.

### Bacteriophage adsorption assay

An adsorption assay was performed according to the protocol described by Jeon and Yong^41^ with minor modifications. Briefly, the mid-log phase of the host bacterial suspension was diluted to an OD_600_ of 0.2 (approximately 10^8^ CFU/mL) in TSB medium and infected with the phage at a multiplicity of infection (MOI) of 0.01 before incubation at 37 °C. At 1, 2, 3, 4, 5, 6, 7, 10, and 20 min post-infection, the mixture was collected and centrifuged immediately. The titers of unabsorbed phages were determined using double-layer plaque assays^46^.

### One-step growth curve analysis

The one-step growth curve was generated as described previously^30^ with some modifications. Briefly, bacterial culture (with an OD_600_ of 0.2, approximately 10^8^ CFU/mL) was infected with phage suspension (10^8^ PFU/mL) to obtain an MOI of 0.001 followed by incubation for 7 min at 37 °C and centrifugation. The pellet was resuspended in 10 mL of TSB medium and cultured at 37 °C with shaking. Aliquots (0.1 mL of the cultures) were taken every 10 min for 2 h and the phage titer was determined via double-layer plaque assays^46^. Countable plaques were calculated as plotted curves to observe the latency period of the phage, and the average burst size of the phage was calculated from the ratio of phage titer at the beginning and end of the experiments.

### Bacterial growth inhibition assay

A bacterial growth inhibition assay was performed in a 96-well microtiter plate as previously described, with slight modifications^21^. In all experiments, mid-log phase *P. aeruginosa* strains in fresh TSB medium at approximately 10^8^ CFU/mL were mixed with phage suspension to obtain MOIs of 0.1, 1, 10 or 100. Plates were incubated at 37 °C and every 1 h for 6 h, bacterial growth was measured absorbance at OD_590_ by using a microplate reader (Molecular Devices, USA). Bacterial cultures without phages were used as the controls. Three independent experiments were performed.

### pH and thermal stability assays

Phage stability tests under different pH conditions were conducted as previously described^29^. Phage VL1 (10^8^ PFU/mL) was incubated separately in SM buffer ranging from pH 1 to 10 at 37 °C for an hour. For thermal stability, phage VL1 was suspended in SM buffer pH 7.4 and then separately incubated at different temperatures including 4 °C (control), 25, 37, 40, 50, 60, 70, and 80 for an hour. Phage titers were then evaluated using the double-agar overlay plaque technique^46^. The experiments were performed in triplicates.

### Bacterial lysis efficiency and EOP determination

Efficiency of bacterial lysis was determined by dropping 10 μL of serially diluted phage suspension onto a lawn of tested bacterial strains (Table 1) and incubated at 37 °C overnight. The appearance of a clear lysis zone was reported as (+) = complete lytic, (T) = turbid lytic, or (−) = non-lytic or resistant. The host cells obtained complete or turbid lytic results were confirmed using double-layer plaque assays^46^ and efficiency of plating (EOP). The EOP value was calculated by dividing the number of plaques on each bacterial strain by the number of plaques conducted on host strain ATCC27853. Following the calculation, EOP is classified as high (EOP ≥ 0.5), moderate (EOP > 0.1– < 0.5), low (EOP ≤ 0.1) and no activity (EOP < 0.001).

### Biofilm inhibition assay

Anti-biofilm activity was determined as previously described with slight modifications^41,47^. *P. aeruginosa* was diluted to an OD_600_ of 0.2 in TSB medium and incubated in 96-well flat-bottomed polystyrene microplates (Corning, USA) at 37 °C for 48 h. After biofilm formation, the wells were rinsed with phosphate-buffered saline (PBS; Sigma, USA) to remove debris. Then, approximately 10^9^ PFU/mL of phage suspension (to obtain MOI of 10) or TSB medium was added to each well and incubated at 37 °C for 6, 12 and 24 h. Subsequently, for crystal violet staining, the plates were stained with 0.1% crystal violet solution (Merck, Germany). To dissolve the stain, 30% (v/v) glacial acetic acid (Merck) was added, and the absorbance was measured at OD_595_. In order to determine the number of viable bacterial cells within the biofilm, the biofilm within the 96-well plate was washed with PBS before adding PBS to the wells. The bottom and walls of the wells were scraped with a loop before vigorous pipetting. The contents of each well were serially diluted in PBS containing 10 mM ferrous ammonium sulfate (Merck, Germany) to inactivate free phages prior to viable bacterial counts. The experiments were carried out independently in triplicate with a duplicate assay.

### Phage therapy assay in a *G. mellonella* infection model

In vivo test was conducted in *G. mellonella* larva as described previously, with some modifications^41^. Larvae weighing 200–250 mg and exhibiting cream color and free of injury were used for experiments. The larvae were divided into the following 4 groups of 10 larvae each and injected with a different solution. For group I (control), larvae injected with 10 µL of PBS (Invitrogen), group II (phage lysate only) larvae injected with 10^4^ or 10^5^ PFU/10 µL of phage VL1, group III (bacterial infection) and group IV (phage treatment) larvae injected with 10 CFU/10 µL of *P. aeruginosa* clinical isolates. At 1 h p.i., the phage VL1 (10^4^ or 10^5^ PFU/10 µL to make the input MOI of 10^3^ or 10^4^ respectively) was injected into the last left-side proleg of the larvae in group IV while other groups were injected with SM buffer. After treatment, the larvae were then incubated in the dark at 37 °C and the number of dead larvae was determined every 12 h for 72 h. The larvae were considered dead when there was no movement in response to touch. The in vivo test was performed three times on separate occasions.

### Whole genome sequencing and bioinformatic analysis

Phage DNA was extracted by extraction of phenol–chloroform as follows^29^. The phage DNA was sent to a commercial facility for sequencing using the Illumina sequencing platform (Macrogen Company, South Korea) and de novo assembly was performed by various k-mers using SPAdes^48^ (version 3.15.0). Analysis of complete genome sequences of phages, including Blastn (megablast) at NCBI GenBank50, was performed to search for highly similar sequences. Pairwise sequence alignments were calculated using EMBOSS Stretcher^49^ to obtain genomic sequence identity. The Easyfig^50^ computer program was used for visual genome comparisons. Analysis of the complete genome sequence of the phage, including prediction of the open reading frame (ORFs), was achieved by rapid annotation using the subsystem technology pipeline (RAST53). The ORF functions were annotated using the protein basic local alignment search tool (Blastp), and protein motif predictions were conducted using the Conserved Domain Search Service (CD search) of the NCBI database. The expressions of the early, middle and late genes cluster of phage VL1 is classified according to the gene organization and expression of the genes similar to N4 phages^21,22^. The termini of the phage genome and the packing mechanism were predicted using PhageTerm^20^. Putative tRNAs in the genome were identified using tRNA Scan-SE^51^. Antimicrobial resistance (AMR) genes and variants were predicted using the Resistance Gene Identifier (RGI) tool incorporated in the Comprehensive Antibiotic Resistance Database (CARD) server^52^. The TAfinder tool was used for the prediction of toxins^53^.

### Construction of phylogenetic tree

Phage complete genome sequence, DNA polymerase, and terminase large subunit genes from *Litunaviruses* were aligned using the MAFFT online server^54^. The resulting alignment was imported into the MEGA X program version 10.2.4, and evolutionary history was inferred using the neighbor-joining method. The robustness of the phylogenetic trees was evaluated using bootstrap analyses based on 1,000 random resamplings.

### Statistical analysis

Statistical analysis of the data was performed using the GraphPad Prism software version 9 (USA). The means were compared using the student’s unpaired *t* test for two-group comparisons and one-way analysis of variance (ANOVA) followed by Dunnett's post hoc test for multi-group comparisons. The log-rank test (Mantel-Cox) was used for survival curves. The significance of the differences between the compared experiments was considered at *P* less than 0.05.

## Supplementary Information

Supplementary Information.

## Data Availability

The complete genomic sequence of phage VL1 was deposited in the NCBI database under accession number OK665488. Data is publicly available now.
