# Supplementary material for: A novel virulent Litunavirus phage possesses therapeutic value against multidrug resistant Pseudomonas aeruginosa
Source: Sci Rep. 2022 Dec 7;12:21193. doi: 10.1038/s41598-022-25576-6 (PMC9729221; doi:10.1038/s41598-022-25576-6)
Supplement: Supplementary file 1 — Supplementary Information. [file 41598_2022_25576_MOESM1_ESM.docx]

**Supplementary information**

**A Novel Virulent *Litunavirus* Phage Possesses Therapeutic Value Against Multidrug Resistant *Pseudomonas aeruginosa***

Varintip Lerdsittikul^1^, Metawee Thongdee^2^, Somjit Chaiwattanarungruengpaisan^2^, Thassanant Atithep^3^, Sukanya Apiratwarrasakul^1^, Patoo Withatanung^4^, Martha R. J. Clokie^5^ & Sunee Korbsrisate^4🖂^

^1^Veterinary Diagnostic Center, Faculty of Veterinary Science, Mahidol University, Nakhon Pathom, Thailand. ^2^The Monitoring Surveillance Center for Zoonotic Diseases in Wildlife and Exotic Animals, Faculty of Veterinary Science, Mahidol University, Nakhon Pathom, Thailand. ^3^Frontier Research Center, Vidyasirimedhi Institute of Science and Technology, Rayong, Thailand. ^4^Department of Immunology, Faculty of Medicine Siriraj Hospital, Mahidol University, Bangkok, Thailand. ^5^Department of Genetics and Genome Biology, University of Leicester, Leicester, United Kingdom.

*Corresponding author: Sunee.kor@mahidol.edu

**
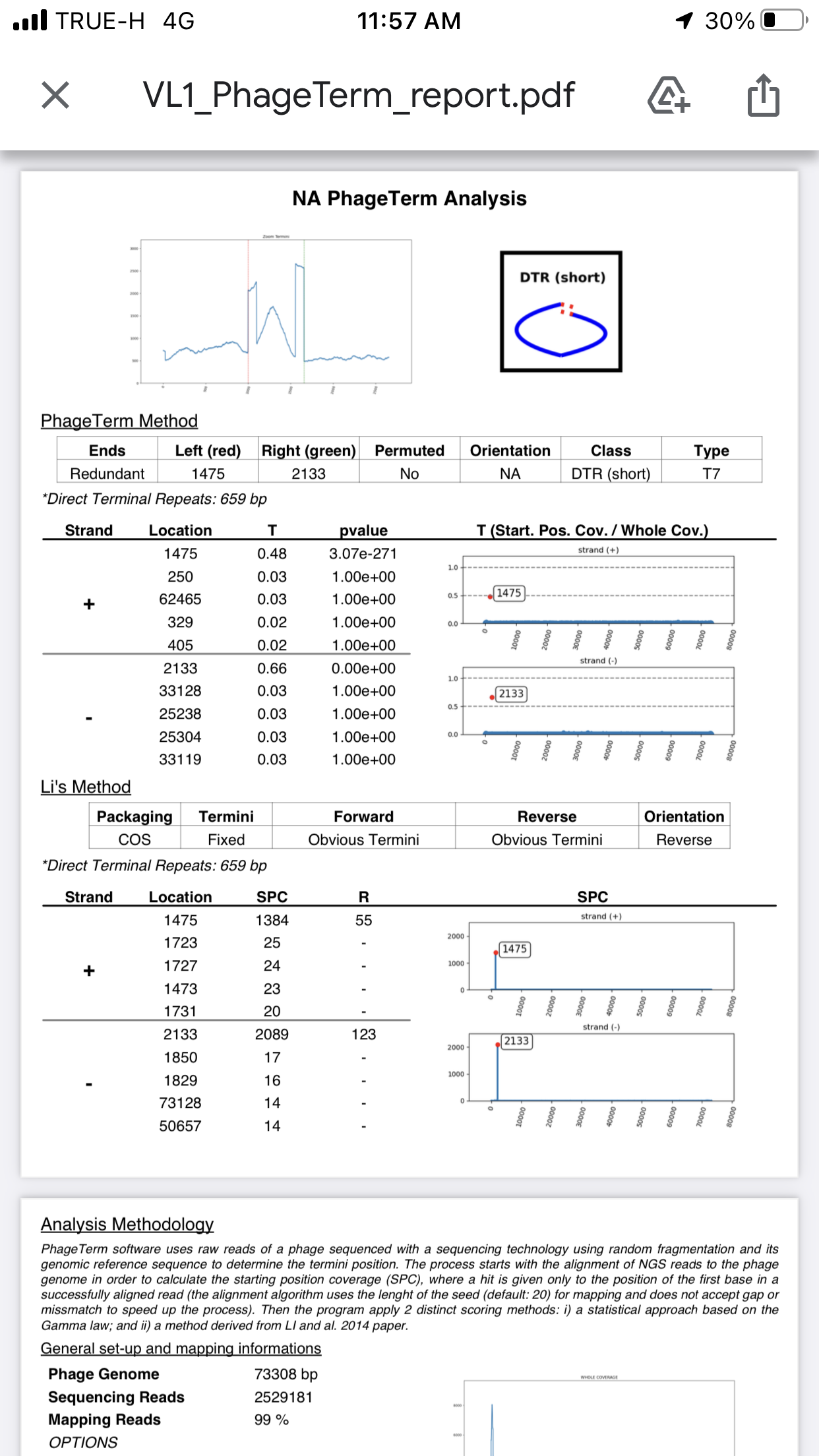
**

**Figure S1.** PhageTerm analysis of vB_PaeS_VL1 genome

**
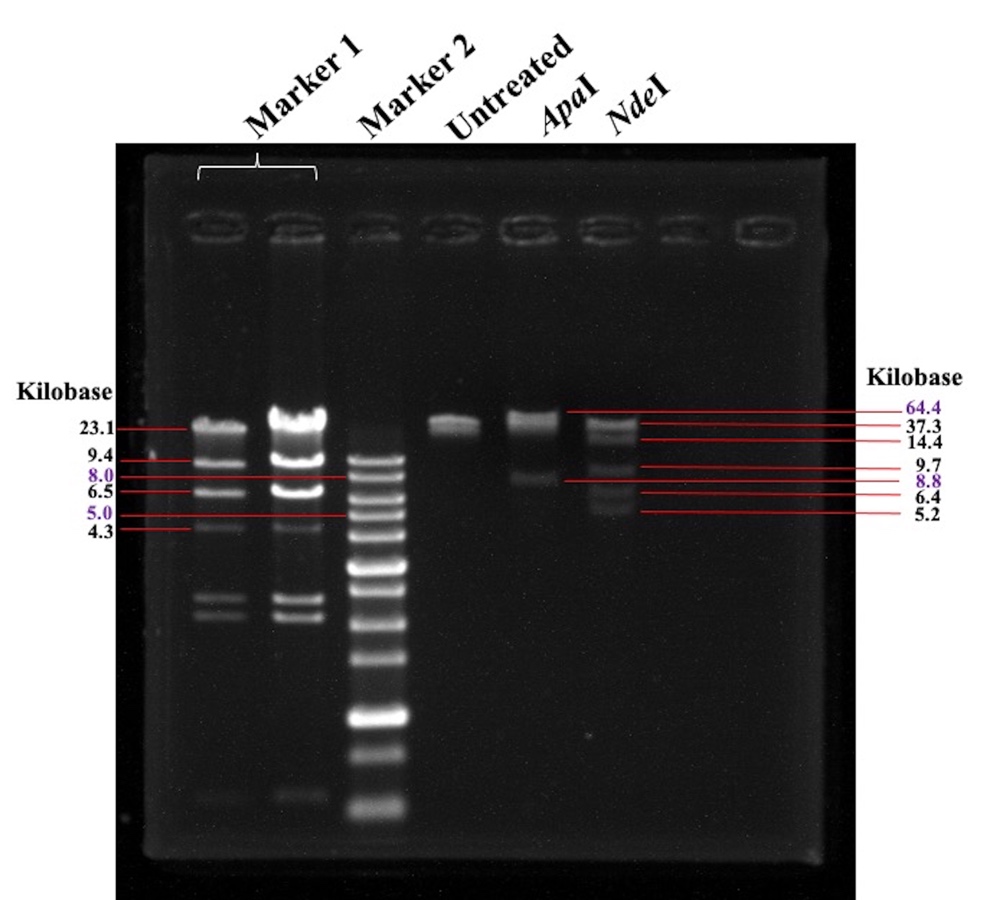
**

**Figure S2.** Restriction digestion profiles of vB_PaeS_VL1 genome. The genome of phage VL1 was digested with *Apa*I and *Nde*I. Marker 1: Lambda DNA/ *Hind*III marker. Marker 2: 1 kb ladder.

**Table S1.** Predicted open reading frame (ORF) in the vB_PaeS_VL1 genome

| **ORF** | **Start** | **Stop** | **Direction** | **Number of residues** | **Molecular**  **Mass**  **(kDa)** | **Calculated**  **Isoelectric**  **point** | **Putative function** | **Conserved protein domain superfamily** |
| --- | --- | --- | --- | --- | --- | --- | --- | --- |
| 1 | 81 | 389 | + | 102 | 11.7 | 8.07 | Hypothetical protein |  |
| 2 | 595 | 1257 | + | 220 | 25.0 | 5.40 | Hypothetical protein |  |
| 3 | 1674 | 1928 | + | 84 | 9.6 | 5.86 | Hypothetical protein |  |
| 4 | 2342 | 2533 | + | 63 | 7.2 | 4.32 | Hypothetical protein |  |
| 5 | 2548 | 2790 | + | 80 | 9.1 | 7.74 | Hypothetical protein |  |
| 6 | 2787 | 3017 | + | 76 | 8.9 | 5.30 | Putative transcriptional regulator |  |
| 7 | 3017 | 3265 | + | 82 | 9.8 | 5.17 | Hypothetical protein |  |
| 8 | 3269 | 3577 | + | 102 | 12.1 | 5.06 | Wall-associated receptor kinase-like 20-like protein |  |
| 9 | 3561 | 3863 | + | 100 | 11.4 | 10.40 | Hypothetical protein |  |
| 10 | 3867 | 4070 | + | 67 | 7.3 | 7.62 | Hypothetical protein |  |
| 11 | 4100 | 4318 | + | 72 | 8.1 | 7.83 | Hypothetical protein |  |
| 12 | 4315 | 4548 | + | 77 | 8.6 | 9.30 | Hypothetical protein |  |
| 13 | 4545 | 4766 | + | 73 | 8.1 | 4.54 | Hypothetical protein |  |
| 14 | 4838 | 5023 | + | 61 | 7.0 | 4.60 | Hypothetical protein |  |
| 15 | 5117 | 5407 | + | 96 | 8.9 | 9.30 | Hypothetical protein |  |
| 16 | 5592 | 5957 | + | 121 | 13.2 | 6.51 | Hypothetical protein |  |
| 17 | 5957 | 6358 | + | 133 | 14.6 | 7.77 | N4 gp14-like protein | PHA00684 (hypothetical protein) |
| 18 | 6355 | 6609 | + | 84 | 9.6 | 5.82 | Transcriptional regulator |  |
| 19 | 6609 | 7049 | + | 146 | 16.2 | 5.72 | Hypothetical protein |  |
| 20 | 7089 | 7436 | + | 115 | 13.3 | 7.87 | Hypothetical protein |  |
| 21 | 7448 | 8380 | + | 310 | 35.9 | 5.77 | RNA polymerase small subunit | RNA_pol super family (DNA-dependent RNA polymerase) |
| 22 | 8393 | 8653 | + | 86 | 9.9 | 4.58 | Hypothetical protein |  |
| 23 | 8944 | 9216 | + | 90 | 10.6 | 9.07 | Hypothetical protein |  |
| 24 | 9251 | 9493 | + | 80 | 9.2 | 4.92 | Putative RNAP2 | RNA_pol super family (DNA-dependent RNA polymerase) |
| 25 | 9800 | 10468 | + | 222 | 25.1 | 9.20 | Hypothetical protein | GIY-YIG_SF super family (GIY-YIG nuclease domain superfamily) |
| 26 | 10491 | 11492 | + | 333 | 37.9 | 4.81 | RNA polymerase large subunit | RNA_pol super family (DNA-dependent RNA polymerase) |
| 27 | 11590 | 11742 | + | 50 | 5.8 | 9.6 | Hypothetical protein |  |
| 28 | 11815 | 12039 | + | 74 | 8.2 | 9.22 | Hypothetical protein |  |
| 29 | 12043 | 12669 | + | 208 | 24.6 | 4.92 | Hypothetical protein |  |
| 30 | 12666 | 13220 | + | 184 | 21.7 | 9.49 | Hypothetical protein |  |
| 31 | 13220 | 13801 | + | 193 | 22.0 | 8.94 | Hypothetical protein |  |
| 32 | 13801 | 14316 | + | 171 | 19.3 | 6.44 | Hypothetical protein |  |
| 33 | 14313 | 14537 | + | 74 | 8.3 | 4.98 | Hypothetical protein |  |
| 34 | 14772 | 14999 | + | 75 | 8.3 | 4.98 | Hypothetical protein |  |
| 35 | 15008 | 15193 | + | 61 | 7.1 | 5.87 | Hypothetical protein |  |
| 36 | 15252 | 16085 | + | 277 | 31.0 | 6.16 | ATP-dependent Clp protease ATP-binding subunit ClpX | P-loop_NTPase super family (P-loop containing Nucleoside Triphosphate Hydrolases) |
| 37 | 16082 | 16324 | + | 80 | 9.1 | 9.36 | Cell division protein |  |
| 38 | 16356 | 17426 | + | 356 | 40.1 | 5.50 | ATPase | MoxR super family  (MoxR-like ATPase) |
| 39 | 17953 | 19179 | + | 408 | 45.6 | 6.77 | Hypothetical protein | DUF2201_N super family  (Putative metallopeptidase) |
| 40 | 19269 | 20435 | + | 388 | 44.1 | 6.23 | RecD-like DNA helicase YrrC | DEAD-like_helicase_N super family  (N-terminal helicase domain of the DEAD-box helicase superfamily) |
| 41 | 20435 | 20962 | + | 175 | 20.0 | 6.84 | Hypothetical protein |  |
| 42 | 20962 | 23577 | + | 871 | 98.0 | 6.12 | Putative DNA polymerase | DNA_pol_A super family  (Family A polymerase primarily fills DNA gaps) |
| 43 | 23710 | 24228 | + | 172 | 19.1 | 7.76 | Putative dCMP deaminase | cytidine_deaminase-like super family  (Cytidine and deoxycytidylate deaminase zinc-binding region) |
| 44 | 24260 | 24400 | + | 46 | 4.9 | 8.05 | Hypothetical protein |  |
| 45 | 24403 | 24801 | + | 132 | 14.3 | 10.35 | Hypothetical protein |  |
| 46 | 24798 | 24989 | + | 63 | 7.2 | 9.66 | HNH endonuclease | HNH_3  (HNH endonuclease) |
| 47 | 24993 | 27509 | + | 838 | 94.1 | 9.04 | Putative rIIA-like protein | HATPase_c  (Histidine kinase-like ATPases) |
| 48 | 27521 | 29299 | + | 592 | 63.6 | 7.17 | rIIB-like protein | Opacity_OapA super family  (Opacity-associated protein A) |
| 49 | 29347 | 29571 | + | 74 | 8.9 | 9.99 | Hypothetical protein | Ribophorin_II super family  (Oligosaccharyltransferase subunit Ribophorin II) |
| 50 | 29655 | 29972 | + | 105 | 11.8 | 7.87 | Hypothetical protein |  |
| 51 | 30022 | 30213 | + | 63 | 7.2 | 4.28 | HNH endonuclease |  |
| 52 | 30206 | 30763 | + | 185 | 21.5 | 6.61 | Hypothetical protein |  |
| 53 | 31338 | 30826 | - | 170 | 18.7 | 6.43 | Hypothetical protein | DUF2514 super family (Protein of unknown function) |
| 54 | 31859 | 31335 | - | 174 | 19.5 | 9.14 | Lysozyme |  |
| 55 | 32050 | 31856 | - | 64 | 7.5 | 4.83 | Hypothetical protein |  |
| 56 | 35560 | 32405 | - | 1051 | 114.1 | 5.18 | Tail fiber protein | PHA00430 super family  (Tail fiber protein) |
| 57 | 36270 | 35599 | - | 223 | 24.8 | 6.89 | Hypothetical protein |  |
| 58 | 36566 | 36267 | - | 99 | 11.4 | 7.98 | Hypothetical protein |  |
| 59 | 37852 | 36563 | - | 429 | 46.4 | 4.8 | Tail fiber protein J | COG4733 super family  (Phage-related protein, tail component) |
| 60 | 38245 | 38673 | + | 142 | 15.8 | 9.72 | Hypothetical protein |  |
| 61 | 38670 | 39044 | + | 124 | 14.2 | 7.7 | Hypothetical protein |  |
| 62 | 39046 | 40056 | + | 336 | 37.8 | 7.57 | Hypothetical protein |  |
| 63 | 40071 | 42239 | + | 722 | 82.2 | 6.01 | Putative DNA primase P4 type | PriCT_1 super family  (Primase C terminal 1) |
| 64 | 42288 | 43022 | + | 244 | 28.1 | 6.10 | N4 gp44-like protein | YlqF_related_GTPase super family  (Circularly permuted YlqF-related GTPases) |
| 65 | 43049 | 43795 | + | 248 | 26.8 | 6.86 | Putative single-stranded DNA-binding protein |  |
| 66 | 43799 | 44164 | + | 121 | 13.9 | 7.76 | Hypothetical protein |  |
| 67 | 44161 | 44604 | + | 147 | 17.2 | 4.8 | N4 gp48-like protein | Phage_gp49_66 super family  (Phage protein N4 Gp49/phage Sf6 gene 66 family) |
| 68 | 44592 | 45152 | + | 186 | 20.4 | 8.66 | N4 gp46-like protein |  |
| 69 | 45164 | 45601 | + | 145 | 14.6 | 4.46 | Hypothetical protein |  |
| 70 | 45659 | 46063 | + | 134 | 14.5 | 5.53 | Hypothetical protein |  |
| 71 | 46468 | 46704 | + | 78 | 8.9 | 6.72 | Hypothetical protein |  |
| 72 | 46701 | 47018 | + | 105 | 11.5 | 5.49 | Hypothetical protein |  |
| 73 | 47028 | 47231 | + | 67 | 7.5 | 9.24 | Hypothetical protein |  |
| 74 | 57443 | 47247 | - | 3398 | 369.9 | 5.16 | Virion assoviated RNA polymerase | olA super family  (Cell envelope integrity inner membrane protein TolA) |
| 75 | 58556 | 57444 | - | 370 | 41.0 | 5.18 | Lytic tail fiber |  |
| 76 | 59007 | 58585 | - | 140 | 15.0 | 5.40 | Lytic tail fiber |  |
| 77 | 59474 | 59007 | - | 155 | 16.7 | 6.21 | Hypothetical protein |  |
| 78 | 61677 | 59455 | - | 740 | 82.2 | 5.40 | Hypothetical protein |  |
| 79 | 62700 | 61735 | - | 321 | 35.1 | 5.68 | Structural protein |  |
| 80 | 63369 | 62704 | - | 221 | 25.0 | 5.02 | Hypothetical protein |  |
| 81 | 64625 | 63426 | - | 399 | 44.0 | 6.21 | Major capsid protein | Capsid_maj_N4  (Major capsid protein) |
| 82 | 65850 | 64660 | - | 396 | 44.5 | 4.43 | Hypothetical protein | ProQ super family  (sRNA-binding protein) |
| 83 | 66188 | 65850 | - | 112 | 13.0 | 5.01 | Hypothetical protein |  |
| 84 | 68438 | 66258 | - | 726 | 81.7 | 4.89 | Putative portal protein |  |
| 85 | 68473 | 68892 | + | 139 | 14.9 | 4.99 | putative dUTPase | Trimeric_dUTPase super family  (Trimeric dUTP diphosphatases) |
| 86 | 68896 | 69195 | + | 99 | 11.1 | 4.50 | Hypothetical protein |  |
| 87 | 69962 | 69228 | - | 244 | 27.9 | 5.52 | N4 gp67-like protein |  |
| 88 | 71611 | 69959 | - | 550 | 63.0 | 5.35 | Putative large terminase  subunit | Terminase_6C super family (Terminase RNaseH-like domain) |
| 89 | 72336 | 71611 | - | 241 | 27.1 | 6.32 | N4 gp69-like protein |  |
| 90 | 72403 | 72834 | + | 143 | 16.0 | 6.29 | Hypothetical protein | P-loop_NTPase super family  (P-loop containing Nucleoside Triphosphate Hydrolases) |
| 91 | 72834 | 73046 | + | 70 | 7.9 | 5.8 | Hypothetical protein |  |
| 92 | 73043 | 73222 | + | 59 | 7.2 | 6.9 | Hypothetical protein |  |

**Table S2** ORFs identified in the genome of *Pseudomonas* phage vB_PaeS_VL1. **Pseudomonas* phages with the highest similarity are indicated.

| **ORF** | **Phage Genus *Litunarvirus**** | Structural Predictions  of phage VL1 | **E-value** | **Percent identity** |
| --- | --- | --- | --- | --- |
| 1 | PA26 | Hypothetical protein | 4e-69 | 100 |
| 2 | vB_Pae575P-3 | Hypothetical protein | 4e-149 | 100 |
| 3 | vB_PaeP_C2-10_Ab09 | Hypothetical protein | 3e-50 | 98 |
| 4 | Pa2 | Hypothetical protein | 8e-39 | 98 |
| 5 | YH6 | Hypothetical protein | 1e-50 | 100 |
| 6 | YH30 | Putative transcriptional regulator | 1e-41 | 86 |
| 7 | Pa2 | Hypothetical protein | 5e-51 | 94 |
| 8 | YH30 | Wall-associated receptor kinase-like 20-like protein | 5e-68 | 97 |
| 9 | LIT1 | Hypothetical protein | 3e-63 | 95 |
| 10 | YH30 | Hypothetical protein | 1e-36 | 100 |
| 11 | YH30 | Hypothetical protein | 1e-42 | 99 |
| 12 | LP14 | Hypothetical protein | 2e-45 | 100 |
| 13 | PA26 | Hypothetical protein | 2e-43 | 100 |
| 14 | DL64 | Hypothetical protein | 1e-35 | 98 |
| 15 | PA26 | Hypothetical protein | 2e-61 | 100 |
| 16 | LIT1 | Hypothetical protein | 6e-17 | 40 |
| 17 | LIT1 | N4 gp14-like protein | 1e-95 | 99 |
| 18 | YH30 | Transcriptional regulator | 4e-52 | 96 |
| 19 | PA26 | Hypothetical protein | 8e-106 | 99 |
| 20 | LP14 | Hypothetical protein | 4e-72 | 91 |
| 21 | YH30 | RNA polymerase small subunit | 0.0 | 99 |
| 22 | LP14 | Hypothetical protein | 3e-54 | 98 |
| 23 | vB_PaeP_C2-10_Ab09 | Hypothetical protein | 3e-59 | 97 |
| 24 | YH6 | Putative RNAP2 | 3e-54 | 100 |
| 25 | LP14 | Hypothetical protein | 8e-165 | 99 |
| 26 | LIT1 | RNA polymerase large subunit | 0.0 | 99 |
| 27 | LP14 | Hypothetical protein | 2e-26 | 92 |
| 28 | vB_PaeP_PYO2 | Hypothetical protein | 5e-41 | 96 |
| 29 | LP14 | Hypothetical protein | 8e-134 | 82 |
| 30 | PA26 | Hypothetical protein | 3e-108 | 83 |
| 31 | LP14 | Hypothetical protein | 6e-139 | 100 |
| 32 | LP14 | Hypothetical protein | 2e-103 | 89 |
| 33 | Pa2 | Hypothetical protein | 4e-45 | 95 |
| 34 | vB_PaeP_MAG4 | Hypothetical protein | 7e-15 | 44 |
| 35 | LP14 | Hypothetical protein | 6e-36 | 98 |
| 36 | DL64 | ATP-dependent Clp protease ATP-binding subunit ClpX | 0.0 | 96 |
| 37 | YH6 | Cell division protein | 2e-52 | 98 |
| 38 | vB_Pae575P-3 | ATPase | 0.0 | 99 |
| 39 | LP14 | Hypothetical protein | 0.0 | 97 |
| 40 | DL64 | RecD-like DNA helicase YrrC | 0.0 | 100 |
| 41 | DL64 | Hypothetical protein | 2e-124 | 99 |
| 42 | LP14 | Putative DNA polymerase | 0.0 | 99 |
| 43 | Pa2 | Putative dCMP deaminase | 3e-116 | 91 |
| 44 | Pa2 | Hypothetical protein | 1e-23 | 98 |
| 45 | LP14 | Hypothetical protein | 4e-88 | 97 |
| 46 | YH6 | HNH endonuclease | 4e-40 | 100 |
| 47 | YH6 | Putative rIIA-like protein | 0.0 | 98 |
| 48 | YH30 | rIIB-like protein | 0.0 | 98 |
| 49 | LP14 | Hypothetical protein | 1e-39 | 88 |
| 50 | LIT1 | Hypothetical protein | 3e-69 | 100 |
| 51 | YH30 | HNH endonuclease | 5e-34 | 94 |
| 52 | PA26 | Hypothetical protein | 2e-131 | 99 |
| 53 | LIT1 | Hypothetical protein | 2e-119 | 100 |
| 54 | YH30 | Lysozyme | 6e-123 | 99 |
| 55 | PA26 | Hypothetical protein | 5e-32 | 91 |
| 56 | YH30 | Tail fiber protein | 0.0 | 81 |
| 57 | YH30 | Hypothetical protein | 2e-162 | 99 |
| 58 | YH30 | Hypothetical protein | 8e-65 | 100 |
| 59 | YH30 | Tail fiber protein J | 0.0 | 100 |
| 60 | LIT1 | Hypothetical protein | 4e-98 | 99 |
| 61 | vB_PaeP_MAG4 | Hypothetical protein | 6e-84 | 95 |
| 62 | Pae575P-3 | Hypothetical protein | 0.0 | 99 |
| 63 | phi176 | Putative DNA primase P4 type | 0.0 | 99 |
| 64 | LIT1 | N4 gp44-like protein | 0.0 | 100 |
| 65 | YH6 | Putative single-stranded DNA-binding protein | 0.0 | 100 |
| 66 | YH30 | Hypothetical protein | 1e-82 | 99 |
| 67 | LIT1 | N4 gp48-like protein | 1e-102 | 99 |
| 68 | LIT1 | N4 gp46-like protein | 7e-137 | 100 |
| 69 | vB_Pae575P-3 | Hypothetical protein | 2e-90 | 100 |
| 70 | LIT1 | Hypothetical protein | 1e-92 | 99 |
| 71 | Pa2 | Hypothetical protein | 3e-47 | 89 |
| 72 | vB_PaeP_C2-10_Ab09 | Hypothetical protein | 3e-70 | 100 |
| 73 | Pa2 | Hypothetical protein | 7e-36 | 100 |
| 74 | YH30 | Virion assoviated RNA polymerase | 0.00 | 99 |
| 75 | YH30 | Lytic tail fiber | 0.00 | 99 |
| 76 | YH30 | Lytic tail fiber | 2e-103 | 99 |
| 77 | LP14 | Hypothetical protein | 6e-107 | 99 |
| 78 | LP14 | Hypothetical protein | 0.0 | 99 |
| 79 | vB_Pae575P-3 | Structural protein | 0.0 | 99 |
| 80 | vB_PaeP_MAG4 | Hypothetical protein | 4e-161 | 100 |
| 81 | YH30 | Major capsid protein | 0.0 | 99 |
| 82 | vB_Pae575P-3 | Hypothetical protein | 0.0 | 96 |
| 83 | LIT1 | Hypothetical protein | 2e-73 | 100 |
| 84 | LP14 | Putative portal protein | 0.00 | 99 |
| 85 | PEV2 | putative dUTPase | 3e-96 | 99 |
| 86 | LIT1 | Hypothetical protein | 3e-66 | 100 |
| 87 | vB_PaeP_C2-10_Ab09 | N4 gp67-like protein | 0.00 | 100 |
| 88 | LP14 | Putative large terminase  subunit | 0.00 | 99 |
| 89 | vB_PaeP_C2-10_Ab09 | N4 gp69-like protein | 2e-179 | 100 |
| 90 | PA26 | Hypothetical protein | 4e-98 | 97 |
| 91 | PA26 | Hypothetical protein | 1e-43 | 99 |
| 92 | YH30 | Hypothetical protein | 4e-32 | 100 |

**Table S3.** Intergenomic similarity of vB_PaeS_VL1 sequences in comparison with 50 top BLASTn hits.

| **Scientific Name** | **Accession number** | **Percent Query Cover** | **Percent identity** |
| --- | --- | --- | --- |
| *Pseudomonas* phage VB_PaeS_VL1 | [OK665488.1](https://www.ncbi.nlm.nih.gov/nucleotide/OK665488.1?report=genbank&log$=nucltop&blast_rank=1&RID=1BRTEV7S01R) | 100 | 100 |
| *Pseudomonas* phage PA26 | [NC_041907.1](https://www.ncbi.nlm.nih.gov/nucleotide/NC_041907.1?report=genbank&log$=nucltop&blast_rank=2&RID=1BRTEV7S01R) | 97 | 97.04 |
| *Pseudomonas* phage vB_PaeP_DEV | [MF490238.1](https://www.ncbi.nlm.nih.gov/nucleotide/MF490238.1?report=genbank&log$=nucltop&blast_rank=3&RID=1BRTEV7S01R) | 95 | 95.78 |
| *Pseudomonas* phage vB_PaeP_PYO2 | [MF490236.1](https://www.ncbi.nlm.nih.gov/nucleotide/MF490236.1?report=genbank&log$=nucltop&blast_rank=4&RID=1BRTEV7S01R) | 95 | 95.77 |
| *Pseudomonas* phage PEV2 | [KU948710.1](https://www.ncbi.nlm.nih.gov/nucleotide/KU948710.1?report=genbank&log$=nucltop&blast_rank=5&RID=1BRTEV7S01R) | 95 | 95.77 |
| *Pseudomonas* phage RWG | [KM411958.1](https://www.ncbi.nlm.nih.gov/nucleotide/KM411958.1?report=genbank&log$=nucltop&blast_rank=6&RID=1BRTEV7S01R) | 95 | 95.77 |
| *Pseudomonas* phage YH30 | [KP994390.1](https://www.ncbi.nlm.nih.gov/nucleotide/KP994390.1?report=genbank&log$=nucltop&blast_rank=7&RID=1BRTEV7S01R) | 96 | 96.33 |
| *Pseudomonas* phage YH6 | [KM974184.1](https://www.ncbi.nlm.nih.gov/nucleotide/KM974184.1?report=genbank&log$=nucltop&blast_rank=8&RID=1BRTEV7S01R) | 96 | 96.28 |
| *Pseudomonas* phage vB_PaeP_C2-10_Ab09 | [HG962375.1](https://www.ncbi.nlm.nih.gov/nucleotide/HG962375.1?report=genbank&log$=nucltop&blast_rank=9&RID=1BRTEV7S01R) | 95 | 95.64 |
| *Pseudomonas aeruginosa* isolate ph_P3P1 genome assembly, chromosome: P3P1 | [LT594787.1](https://www.ncbi.nlm.nih.gov/nucleotide/LT594787.1?report=genbank&log$=nucltop&blast_rank=10&RID=1BRTEV7S01R) | 94 | 95.57 |
| *Pseudomonas* phage vB_PaeP_TUMS_P121 | [MZ955867.1](https://www.ncbi.nlm.nih.gov/nucleotide/MZ955867.1?report=genbank&log$=nucltop&blast_rank=11&RID=1BRTEV7S01R) | 97 | 95.39 |
| *Pseudomonas* phage LP14 | [MH356729.1](https://www.ncbi.nlm.nih.gov/nucleotide/MH356729.1?report=genbank&log$=nucltop&blast_rank=12&RID=1BRTEV7S01R) | 96 | 95.95 |
| *Pseudomonas* phage vB_PaeS_TUMS_P81 | [OL519844.1](https://www.ncbi.nlm.nih.gov/nucleotide/OL519844.1?report=genbank&log$=nucltop&blast_rank=13&RID=1BRTEV7S01R) | 97 | 95.37 |
| *Pseudomonas* phage DL64 | [KR054032.1](https://www.ncbi.nlm.nih.gov/nucleotide/KR054032.1?report=genbank&log$=nucltop&blast_rank=14&RID=1BRTEV7S01R) | 95 | 95.88 |
| *Pseudomonas* phage phi176 | [KM411960.1](https://www.ncbi.nlm.nih.gov/nucleotide/KM411960.1?report=genbank&log$=nucltop&blast_rank=15&RID=1BRTEV7S01R) | 96 | 95.1 |
| *Pseudomonas* phage Pa2 | [NC_027345.1](https://www.ncbi.nlm.nih.gov/nucleotide/NC_027345.1?report=genbank&log$=nucltop&blast_rank=16&RID=1BRTEV7S01R) | 96 | 95.03 |
| *Pseudomonas* phage LY218 | [MN906996.1](https://www.ncbi.nlm.nih.gov/nucleotide/MN906996.1?report=genbank&log$=nucltop&blast_rank=17&RID=1BRTEV7S01R) | 96 | 95 |
| *Pseudomonas* phage vB_Pae1396P-5 | [KX171210.1](https://www.ncbi.nlm.nih.gov/nucleotide/KX171210.1?report=genbank&log$=nucltop&blast_rank=18&RID=1BRTEV7S01R) | 96 | 95.68 |
| *Pseudomonas* phage vB_Pae575P-3 | [KX171209.1](https://www.ncbi.nlm.nih.gov/nucleotide/KX171209.1?report=genbank&log$=nucltop&blast_rank=19&RID=1BRTEV7S01R) | 96 | 95.74 |
| *Pseudomonas* phage vB_PaeP_MAG4 | [KR052142.1](https://www.ncbi.nlm.nih.gov/nucleotide/KR052142.1?report=genbank&log$=nucltop&blast_rank=20&RID=1BRTEV7S01R) | 95 | 96.04 |
| *Pseudomonas* phage LIT1 | [FN422399.1](https://www.ncbi.nlm.nih.gov/nucleotide/FN422399.1?report=genbank&log$=nucltop&blast_rank=21&RID=1BRTEV7S01R) | 95 | 95.28 |
| *Pseudomonas* phage LUZ7 | [FN422398.1](https://www.ncbi.nlm.nih.gov/nucleotide/FN422398.1?report=genbank&log$=nucltop&blast_rank=22&RID=1BRTEV7S01R) | 18 | 75.49 |
| *Pseudomonas* phage vB_PaeS_TUMS_P6 | [OL519842.1](https://www.ncbi.nlm.nih.gov/nucleotide/OL519842.1?report=genbank&log$=nucltop&blast_rank=23&RID=1BRTEV7S01R) | 19 | 73.82 |
| *Pseudomonas* phage KPP21 | [LC064302.1](https://www.ncbi.nlm.nih.gov/nucleotide/LC064302.1?report=genbank&log$=nucltop&blast_rank=24&RID=1BRTEV7S01R) | 17 | 76 |
| *Pseudomonas* phage vB_Pae_AM.P2 | [MT416090.1](https://www.ncbi.nlm.nih.gov/nucleotide/MT416090.1?report=genbank&log$=nucltop&blast_rank=25&RID=1BRTEV7S01R) | 17 | 75.89 |
| *Pseudomonas* phage vB_PA14 | [MT496753.1](https://www.ncbi.nlm.nih.gov/nucleotide/MT496753.1?report=genbank&log$=nucltop&blast_rank=26&RID=1BRTEV7S01R) | 1 | 99.35 |
| *Pseudomonas* phage vB_PA15 | [MT496754.1](https://www.ncbi.nlm.nih.gov/nucleotide/MT496754.1?report=genbank&log$=nucltop&blast_rank=27&RID=1BRTEV7S01R) | 1 | 78.54 |
| *Alteromonas* sp. RKMC-009 | [CP031010.1](https://www.ncbi.nlm.nih.gov/nucleotide/CP031010.1?report=genbank&log$=nucltop&blast_rank=28&RID=1BRTEV7S01R) | 1 | 75.03 |
| *Pectobacterium* phage Nepra | [NC_048704.1](https://www.ncbi.nlm.nih.gov/nucleotide/NC_048704.1?report=genbank&log$=nucltop&blast_rank=29&RID=1BRTEV7S01R) |  | 73.79 |
| *Pectobacterium* phage vB_PatP_CB3 | [KY514265.1](https://www.ncbi.nlm.nih.gov/nucleotide/KY514265.1?report=genbank&log$=nucltop&blast_rank=30&RID=1BRTEV7S01R) | 1 | 73.84 |
| *Pectobacterium* phage vB_PatP_CB1 | [NC_048653.1](https://www.ncbi.nlm.nih.gov/nucleotide/NC_048653.1?report=genbank&log$=nucltop&blast_rank=31&RID=1BRTEV7S01R) | 1 | 73.84 |
| *Pectobacterium* phage vB_PatP_CB4 | [NC_048654.1](https://www.ncbi.nlm.nih.gov/nucleotide/NC_048654.1?report=genbank&log$=nucltop&blast_rank=32&RID=1BRTEV7S01R) | 1 | 73.75 |
| *Pectobacterium* phage phiA41 | [NC_048659.1](https://www.ncbi.nlm.nih.gov/nucleotide/NC_048659.1?report=genbank&log$=nucltop&blast_rank=33&RID=1BRTEV7S01R) | 1 | 73.96 |
| *Pectobacterium* phage A38 | [KY083726.1](https://www.ncbi.nlm.nih.gov/nucleotide/KY083726.1?report=genbank&log$=nucltop&blast_rank=34&RID=1BRTEV7S01R) | 1 | 73.96 |
| *Kosakonia* phage Kc283 | [MZ348421.1](https://www.ncbi.nlm.nih.gov/nucleotide/MZ348421.1?report=genbank&log$=nucltop&blast_rank=35&RID=1BRTEV7S01R) | 1 | 73.27 |
| *Pectobacterium* phage Horatius | [MN812691.1](https://www.ncbi.nlm.nih.gov/nucleotide/MN812691.1?report=genbank&log$=nucltop&blast_rank=36&RID=1BRTEV7S01R) | 1 | 73.26 |
| P*ectobacterium* phage Possum | [MN812687.1](https://www.ncbi.nlm.nih.gov/nucleotide/MN812687.1?report=genbank&log$=nucltop&blast_rank=37&RID=1BRTEV7S01R) | 1 | 73.26 |
| *Pseudomonas* phage Littlefix | [NC_048697.1](https://www.ncbi.nlm.nih.gov/nucleotide/NC_048697.1?report=genbank&log$=nucltop&blast_rank=38&RID=1BRTEV7S01R) | 1 | 84.24 |
| *Pseudomonas* phage 98PfluR60PP | [MH179480.1](https://www.ncbi.nlm.nih.gov/nucleotide/MH179480.1?report=genbank&log$=nucltop&blast_rank=39&RID=1BRTEV7S01R) | 0 | 81.33 |
| *Achromobacter* phage vB_AxyP_19-32_Axy12 | [NC_054968.1](https://www.ncbi.nlm.nih.gov/nucleotide/NC_054968.1?report=genbank&log$=nucltop&blast_rank=40&RID=1BRTEV7S01R) | 0 | 72.9 |
| *Achromobacter* phage vB_AxyP_19-32_Axy04 | [NC_054967.1](https://www.ncbi.nlm.nih.gov/nucleotide/NC_054967.1?report=genbank&log$=nucltop&blast_rank=41&RID=1BRTEV7S01R) | 0 | 72.9 |
| *Virgibacillus necropolis* | [CP022437.1](https://www.ncbi.nlm.nih.gov/nucleotide/CP022437.1?report=genbank&log$=nucltop&blast_rank=42&RID=1BRTEV7S01R) | 0 | 100 |
| *Halopseudomonas litoralis* | [LT629748.1](https://www.ncbi.nlm.nih.gov/nucleotide/LT629748.1?report=genbank&log$=nucltop&blast_rank=43&RID=1BRTEV7S01R) | 0 | 95.56 |
| Prokaryotic dsDNA virus sp. | [MK892634.1](https://www.ncbi.nlm.nih.gov/nucleotide/MK892634.1?report=genbank&log$=nucltop&blast_rank=44&RID=1BRTEV7S01R) | 0 | 92 |
| *Achromobacter* phage vB_AxyP_19-32_Axy13 | [MK962632.1](https://www.ncbi.nlm.nih.gov/nucleotide/MK962632.1?report=genbank&log$=nucltop&blast_rank=45&RID=1BRTEV7S01R) | 0 | 90.57 |
| *Pseudomonas* phage inbricus | [NC_054970.1](https://www.ncbi.nlm.nih.gov/nucleotide/NC_054970.1?report=genbank&log$=nucltop&blast_rank=46&RID=1BRTEV7S01R) | 0 | 90.57 |
| *Pseudomonas* sp. MSPm1 | [CP059139.1](https://www.ncbi.nlm.nih.gov/nucleotide/CP059139.1?report=genbank&log$=nucltop&blast_rank=47&RID=1BRTEV7S01R) | 0 | 97.56 |
| *Pseudomonas protegens* | [CP013184.1](https://www.ncbi.nlm.nih.gov/nucleotide/CP013184.1?report=genbank&log$=nucltop&blast_rank=48&RID=1BRTEV7S01R) | 0 | 95.35 |
| *Myoviridae* sp. | [BK031590.1](https://www.ncbi.nlm.nih.gov/nucleotide/BK031590.1?report=genbank&log$=nucltop&blast_rank=49&RID=1BRTEV7S01R) | 0 | 95.35 |
| *Pseudomonas aeruginosa* | [CP072783.1](https://www.ncbi.nlm.nih.gov/nucleotide/CP072783.1?report=genbank&log$=nucltop&blast_rank=50&RID=1BRTEV7S01R) | 0 | 95.35 |

**Table S4.** Global genome comparison of vB_PaeS_VL1 and related phages included in this study. The percentage of identity obtained from pairwise genome alignments by EMBOSS Needle tool. ND = not determined or no information.

| **Phage name**  **and**  **Accession number** | **Genome size**  **(bp)** | **ORFs** | **GC content**  **(%)** | **Identity**  **(%)** | **Morphology classification** |
| --- | --- | --- | --- | --- | --- |
| vB_PaeS_VL1  (OK665488.1) | 73308 | 92 | 54.70 | 100 | Podovirus |
| YH6  (KM974184.1) | 73050 | 90 | 54.88 | 93.1 | Podovirus |
| PA26  (NC_041907.1) | 72321 | 88 | 54.82 | 89.1 | Myovirus |
| vB_PaeP_C2-10_Ab09  (HG962375.1) | 72028 | 83 | 54.90 | 87.4 | Podovirus |
| vB_Pae1396P-5  (KX171210.1) | 72508 | 89 | 54.72 | 87.4 | Podovirus |
| vB_Pae575P-3  (KX171209.1) | 72728 | 89 | 54.72 | 87.1 | Podovirus |
| vB_PaeP_MAG4  (NC_031104.1) | 72979 | 94 | 54.8 | 86.8 | Myovirus |
| vB_PaeP_DEV  ([MF490238.1](https://www.ncbi.nlm.nih.gov/nucleotide/MF490238.1?report=genbank&log$=nucltop&blast_rank=2&RID=RPZNPHMJ013)) | 72697 | 91 | 54.9 | 86.7 | Podovirus |
| vB_PaeP_PYO2  ([MF490236.1](https://www.ncbi.nlm.nih.gov/nucleotide/MF490236.1?report=genbank&log$=nucltop&blast_rank=3&RID=RPZNPHMJ013)) | 72697 | 91 | 54.8 | 86.7 | Podovirus |
| PEV2  (KU948710.1) | 72697 | 92 | 54.9 | 86.6 | Podovirus |
| RWG  ([KM411958.1](https://www.ncbi.nlm.nih.gov/nucleotide/KM411958.1?report=genbank&log$=nucltop&blast_rank=5&RID=RPZNPHMJ013)) | 72646 | 94 | 54.87 | 86.6 | ND |
| LIT1  (FN422399.1) | 72544 | 90 | 55 | 86.5 | Podovirus |
| LY218  ([MN906996.1](https://www.ncbi.nlm.nih.gov/nucleotide/MN906996.1?report=genbank&log$=nucltop&blast_rank=14&RID=RPZNPHMJ013)) | 73083 | 88 | 54.9 | 86.5 | ND |
| phi176  ([KM411960.1](https://www.ncbi.nlm.nih.gov/nucleotide/KM411960.1?report=genbank&log$=nucltop&blast_rank=12&RID=RPZNPHMJ013)) | 73048 | 93 | 54.9 | 86.4 | ND |
| Pa2  ([NC_027345.1](https://www.ncbi.nlm.nih.gov/nucleotide/NC_027345.1?report=genbank&log$=nucltop&blast_rank=13&RID=RPZNPHMJ013)) | 73008 | 95 | 54.9 | 86.3 | ND |
| YH30  ([KP994390.1](https://www.ncbi.nlm.nih.gov/nucleotide/KP994390.1?report=genbank&log$=nucltop&blast_rank=6&RID=RPZNPHMJ013)) | 72192 | 86 | 54.92 | 53.4 | Podovirus |
| LP14  ([MH356729.1](https://www.ncbi.nlm.nih.gov/nucleotide/MH356729.1?report=genbank&log$=nucltop&blast_rank=10&RID=RPZNPHMJ013)) | 73080 | 93 | 55 | 48.9 | Podovirus |
| DL64  (KR054032.1) | 72378 | 90 | 55 | 48.9 | Podovirus |
| vB_PaeP_TUMS_P121  (MZ955867.1) | 73001 | 90 | ND | 48.9 | ND |
| vB_PaeS_TUMS_P81  (OL519844.1) | 73167 | 93 | 55 | 48.6 | ND |
